# Enhancing Chemical Stability and Molecular Selectivity of Porous Organic Cages via Core–Shell Polymer Coating

**DOI:** 10.1002/advs.202521917

**Published:** 2026-01-27

**Authors:** Danyu Li, Yanling Huang, Huiyu Liu, Yuzhen Wen, Dongxu Wang, Tao Li, Shan Jiang

**Affiliations:** ^1^ School of Physical Science and Technology ShanghaiTech University Shanghai China; ^2^ School of Physics, Chemistry and Earth Sciences University of Adelaide Adelaide Australia

**Keywords:** core–shell nanostructures, gas selectivity, interfacial engineering, molecular separation, porous organic cages

## Abstract

Porous organic cages (POCs) have emerged as promising molecular materials for gas separation and storage due to their discrete, shape‐persistent structures and accessible cavities. However, their practical application remains limited by intrinsic instability under harsh chemical environments and difficulties in processing. Here, we report a rapid and efficient interfacial strategy for the fabrication of POCs@polymer core–shell nanostructures that exhibit improved chemical robustness and molecular selectivity. Through chiral self‐assembly of enantiomeric cage precursors, well‐defined racemic POC particles are synthesized. A non‐solvent‐induced surface‐aimed polymerization (NISAP) technique enables the formation of dense, conformal polyamic acid (PAA) or polyimide (PI) coatings in a single step, yielding stable, monodisperse core–shell nanostructures. These polymer shells confer exceptional acid resistance upon POC particles, as confirmed by etching and PXRD analyses, and significantly alter their pore environments, leading to enhanced selectivity in gas and vapor separation. Notably, the PAA‐coated materials achieve a CO_2_/N_2_ IAST selectivity of 299.5 and a *para*‐xylene (PX) / *ortho*‐xylene (OX) selectivity of 11.3, which is a tenfold and fourfold improvement over the uncoated material, respectively. Our results offer a general pathway to robust hybrid molecular materials, opening new avenues for advanced separations under demanding industrial conditions.

## Graphical abstract

This study presents an interfacial method to fabricate POC@polymer core–shell nanostructures combining cage porosity with polymer robustness. Through chiral self‐assembly and non‐solvent‐induced surface polymerisation, uniform ∼20 nm polymer‐coated particles are produced. The shells improve chemical resistance and selectivity, offering a scalable platform for advanced separations in harsh environments.

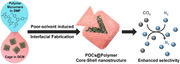

## 1 Introduction

Porous materials have played a central role in advancing technologies related to gas storage, separation, catalysis, and sensing [1, 2]. Over the past two decades, the development of crystalline porous frameworks such as zeolites, metal‐organic frameworks (MOFs), and covalent organic frameworks (COFs) has revolutionized this field, offering unprecedented surface areas and highly tunable pore environments [3, 4]. Despite their high crystallinity and porosity, many of these materials suffer from intrinsic limitations, including poor mechanical flexibility, brittleness, and poor solution processability. These challenges hinder their practical integration into industrial separation systems, flexible devices, and other real‐world applications that demand robust and processable materials [5, 6].

To address these fundamental challenges, researchers have developed several complementary strategies. One widely adopted approach involves blending porous materials with soft polymer matrices to form hybrid composites that combine the porosity of the porous material with the mechanical properties and processability of the polymer [7, 8, 9]. Alternatively, the fabrication of core–shell nanostructures offers a more elegant approach, enabling the integration of multiple functionalities within hierarchically organized architectures where both core and shell layers contribute distinct properties [10, 11]. Through careful design of composition, structure, and interfacial compatibility, such multifunctional materials can achieve enhanced chemical stability [12], improved processability [13], superior dispersibility [14], and tailored selectivity [15] compared to their individual components.

In parallel, the field of porous molecular materials has gained increasing attention. Among them, porous organic cages (POCs)—discrete, shape‐persistent molecules with well‐defined internal cavities—represent a compelling subclass of porous materials with unique properties [16, 17, 18]. Unlike extended frameworks, POCs retain molecular solubility and offer modular synthetic tunability, allowing them to be processed in solution and readily functionalized [19, 20]. Remarkably, some POCs exhibit porosity levels comparable to MOFs and COFs, yet differ fundamentally in their design principles [21, 22]. Unlike the design of crystalline frameworks is guided by the principles of reticular chemistry, the design of molecular cages relies on the precise prediction of intramolecular covalent bonding and intermolecular non‐covalent interactions [23, 24, 25]. However, the nature of molecular crystals also introduces challenges‐namely, a tendency to collapse upon solvent removal, reduced mechanical stability, and poor resistance to chemical environments.

To overcome these obstacles, hybridizing POCs with polymeric materials offers a rational pathway to stabilize their structure and extend their functionality [26, 27]. While embedding cages within polymer matrices can potentially improve their chemical stability, it also severely hinders the mass transport of guest molecules. A more sensible and elegant approach is to fabricate a core–shell POCs@polymer nanostructure featuring a defect‐free polymer coating on the surface of POC particles. The polymer shell can shield the fragile cage core from aggressive chemicals, thereby enhancing its chemical stability without compromising pore accessibility. However, there is no existing method for fabricating well‐defined POC@polymer core–shell particles.

**SCHEME 1:**
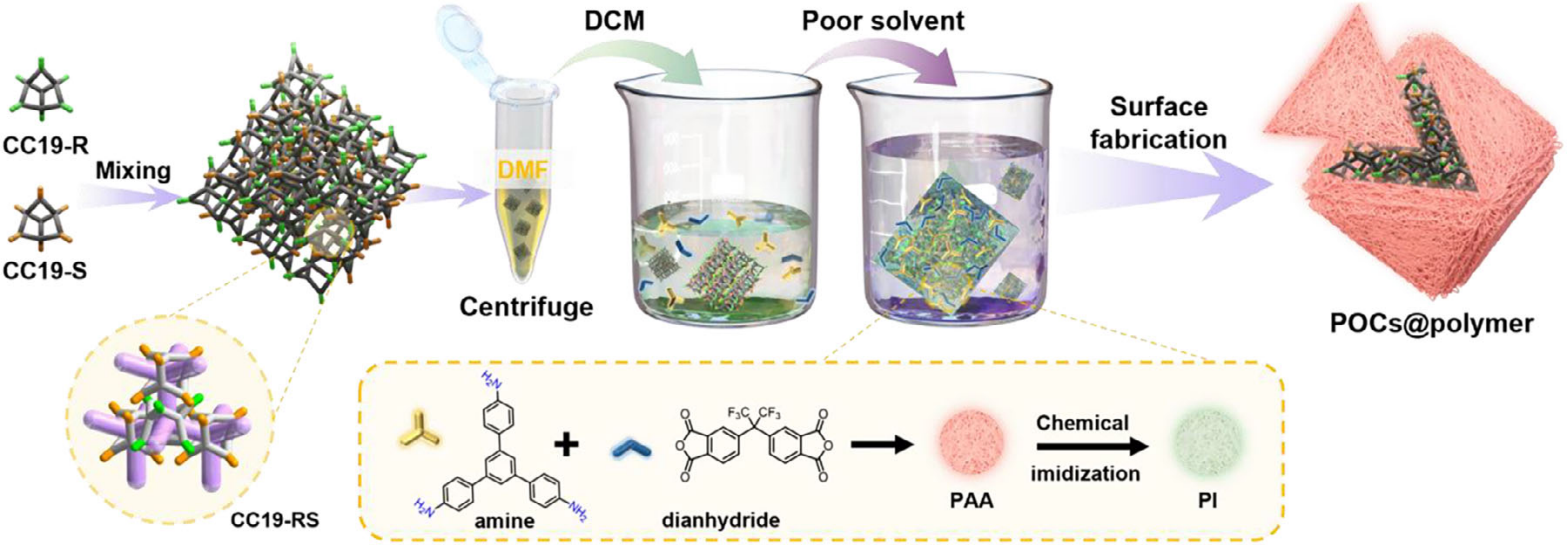
Schematic illustration of the interfacial fabrication of POCs@polymer core–shell nanostructures via NISAP. A solution of enantiomeric porous organic cages (*R*‐ and *S*‐POCs) in DCM is mixed to induce chiral self‐assembly into uniform *RS*‐type particles. The NISAP method induces phase separation by adding a poor solvent, which precipitates amine and dianhydride monomers onto the cage surfaces, thus rapidly forming a nonporous surface polymer layer of PAA on the cages. A subsequent chemical imidization step converts PAA into chemically robust PI, yielding stable POCs@polymer core–shell nanostructures with enhanced protection and functionality. Molecular structures of the cage components and polymer precursors are shown.

Here, we report a simple, rapid, and highly efficient interfacial approach for constructing POCs@polymer core–shell nanostructures with precisely controlled morphology and enhanced stability and selectivity. Our methodology begins with the formation of well‐defined cage particles through controlled mixing of enantiomeric *R*‐ and *S*‐POC solutions, exploiting chiral recognition phenomena to induce predictable self‐assembly into uniform *RS*‐type particles with tunable size and morphology. Following particle formation, we implement a non‐solvent‐induced surface‐aimed polymerization (NISAP) [28] technique that enables single‐step polymer shell formation with remarkable efficiency and control. NISAP operates under mild reaction conditions that preserve the structural integrity of fragile cage molecules, exhibits exceptional surface selectivity while minimizing bulk polymer formation, and allows precise control over shell thickness through simple adjustment of monomer concentration and reaction time. In this process, carefully controlled phase separation is triggered through strategic non‐solvent addition, promoting rapid and selective deposition of amine and dianhydride monomers onto cage surfaces. The subsequent in situ polymerization generates a uniform polyamic acid (PAA) precursor layer, which is then converted to chemically robust polyimide (PI) through brief chemical imidization (Scheme 1). The resulting POC@Polymer nanostructures exhibit enhanced performance metrics, surpassing those of uncoated systems. Chemical stability tests demonstrate resistance to harsh environments, with materials retaining full crystallinity after exposure to 6 M sulfuric acid. Separation performance shows dramatic enhancement, achieving CO_2_/N_2_ IAST selectivity of 299.5 and para‐xylene (PX) / ortho‐xylene (OX) selectivity of 11.3, substantially outperforming uncoated cages.

These hybrid core–shell nanostructures establish a new materials platform that addresses fundamental limitations of molecular porous materials while preserving their distinctive advantages. This approach bridges the gap between laboratory‐scale molecular design and industrial implementation requirements, opening pathways for next‐generation separation technologies based on molecularly engineered hybrid materials [29, 30].

## 2 Results and Discussion

### 2.1 Synthesis of POCs@Polymer Core–Shell Nanostructures

**FIGURE 1:**
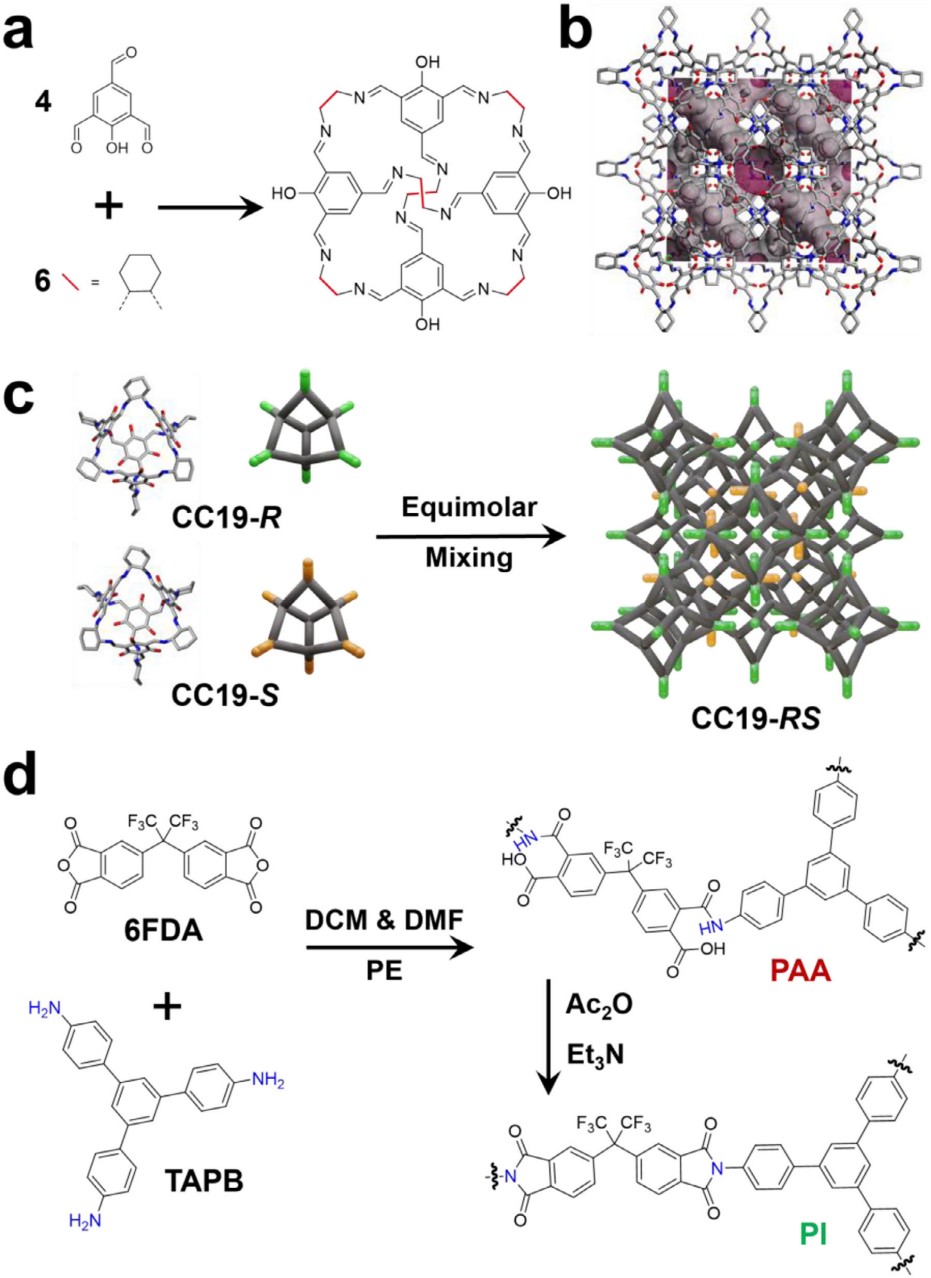
(a) Schematic illustration of the imine condensation reaction between 2‐hydroxy‐1,3,5‐benzenetricarbaldehyde (OH‐TFB) and either (R, R)‐ or (S, S)‐cyclohexane diamine (CHDA) to yield the chiral CC19‐*R* and CC19‐*S* cages. (b) Crystal structure of CC19‐*R*, showing the window‐to‐window packing motif and disordered hydroxyl groups at the cage windows forming a 3D diamondoid porous network. (c) Self‐assembly of enantiomeric CC19‐*R* and CC19‐*S* into racemic CC19‐*RS* cage particles through chiral recognition. (d) The chemical structures of the dianhydride monomer (6FDA) and the triamine monomer (TAPB) are shown. These monomers were used for interfacial polymerization to synthesize polyamic acid (PAA), followed by a chemical imidization process that converted to polyimide (PI).

To start, we synthesized cage molecules CC19‐*R* and CC19‐*S* based on our previous works [15]. Specifically, the analogous [4+6] cage molecule CC19‐*R*, was prepared by reacting 2‐hydroxy‐1,3,5‐benzenetricarbaldehyde (OH‐TFB) with (*R*,*R*)‐1,2‐diaminocyclohexane (*R*,*R*‐CHDA) through an imine condensation reaction, while the opposite cage enantiomer CC19‐*S* was formed by replacing *R*,*R*‐CHDA with (*S*,*S*)‐1,2‐diaminocyclohexane (*S*,*S*‐CHDA) (Figure 1a). These two cage molecules were fully characterized using ^1^H NMR spectroscopy as shown in Figures S1 and S2. In the CC19 homochiral crystal form, CC19‐*R* packs in a window‐to‐window fashion with disordered hydroxyl groups occupying the four cage windows (Figure 1b), creating 3D diamondoid pores connected through the internal cage voids, with a Brunauer‐Emmett‐Teller (BET) surface area (SA_BET_) of 514 m^2^/g [15].

Racemic CC19‐*RS* cage particles were fabricated by simply mixing the corresponding *R* and *S* solutions, utilizing the lower solubility product of the racemic or quasiracemic materials (Figure 1c) [31]. By Powder X‐ray diffraction (PXRD) analysis (Figure S3) confirmed that the racemic cages crystallized in a packing mode identical to that of the monochiral CC19‐*R* and CC19‐*S* forms. The racemic particles adopted uniform octahedral morphologies, with their size tunable from 350 nm to 1.2 µm by adjusting the mixing temperature and reaction time (Table S1), as evidenced by dynamic light scattering (DLS) (Figure S4) and scanning electron microscopy (SEM) (Figure S5).

To achieve uniform core–shell architectures, we employed the NISAP method adapted from our previous studies [28]. We selected anhydride‐amine coupling chemistry for its rapid kinetics, high efficiency, and minimal side reactions. PAA and PI were chosen as shell materials due to their established chemical, thermal, and mechanical stability, versatile functionalization capabilities, and excellent interfacial compatibility with organic molecular cages.

The preparation protocol of POCs@polymer core–shell materials involves the following steps, as shown in Scheme 1. The racemic cage particles were first infiltrated in dimethylformamide (DMF) for a couple of hours and then centrifuged. The purpose of this DMF treatment is to ensure thorough infiltration and prevent agglomeration of the particles. Stock solutions of 4,4'‐(hexafluoroisopropylidene)diphthalic anhydride (6FDA) in dichloromethane (DCM) and 1,3,5‐tris(4‐aminophenyl)benzene (TAPB) in DMF were prepared separately. Predetermined ratios of these monomer solutions were added to cage particles dispersions in DCM, followed by excess petroleum ether as a non‐solvent. The superior solubility of 6FDA and TAPB in DMF drives their preferential partitioning into the DMF phase upon phase separation with petroleum ether. This segregation creates microscale domains with dramatically elevated monomer concentrations, facilitating rapid anhydride‐amine nucleation and crosslinking at hydrophilic cage surfaces. Uniform polymer coatings form within 30 min, yielding CC19@PAA core–shell particles. Chemical imidization using acetic anhydride as a dehydrating agent converts PAA to PI, producing CC19@PI materials (Figure 1d).

To optimize coating uniformity and completeness, we systematically investigated polymer shell morphology across different calculated loading ratios (denoted as CC19@Polymer‐x wt.%, x = W_Polymer_/W_Cage_×100). Transmission electron microscopy (TEM) images revealed that after 6 h of etching with 6 M hydrochloric acid, the chemically unstable cage completely decomposed while the polymer shell remained intact (Figure S6). Complete surface coverage was achieved at 30 wt.% loading, whereas 15 wt.% resulted in incomplete coating. Higher loadings (40–50 wt.%) promoted excessive oligomer formation, compromising coating uniformity (Figure S7). Additionally, smaller cage particles (700 nm) exhibited superior coating efficiency compared to larger analogues (2 µm), with reduced surface oligomer formation as shown in SEM images (Figure S8). Based on these optimization studies, we employed CC19‐*RS* particles (700 nm) as core materials with 6FDA and TAPB monomers at 30 wt.% calculated loading to prepare CC19@polymer‐30 wt.% materials for subsequent characterization and application studies.

### 2.2 Structural Characterisation of POCs@Polymer Core–Shell Nanostructures

**FIGURE 2:**
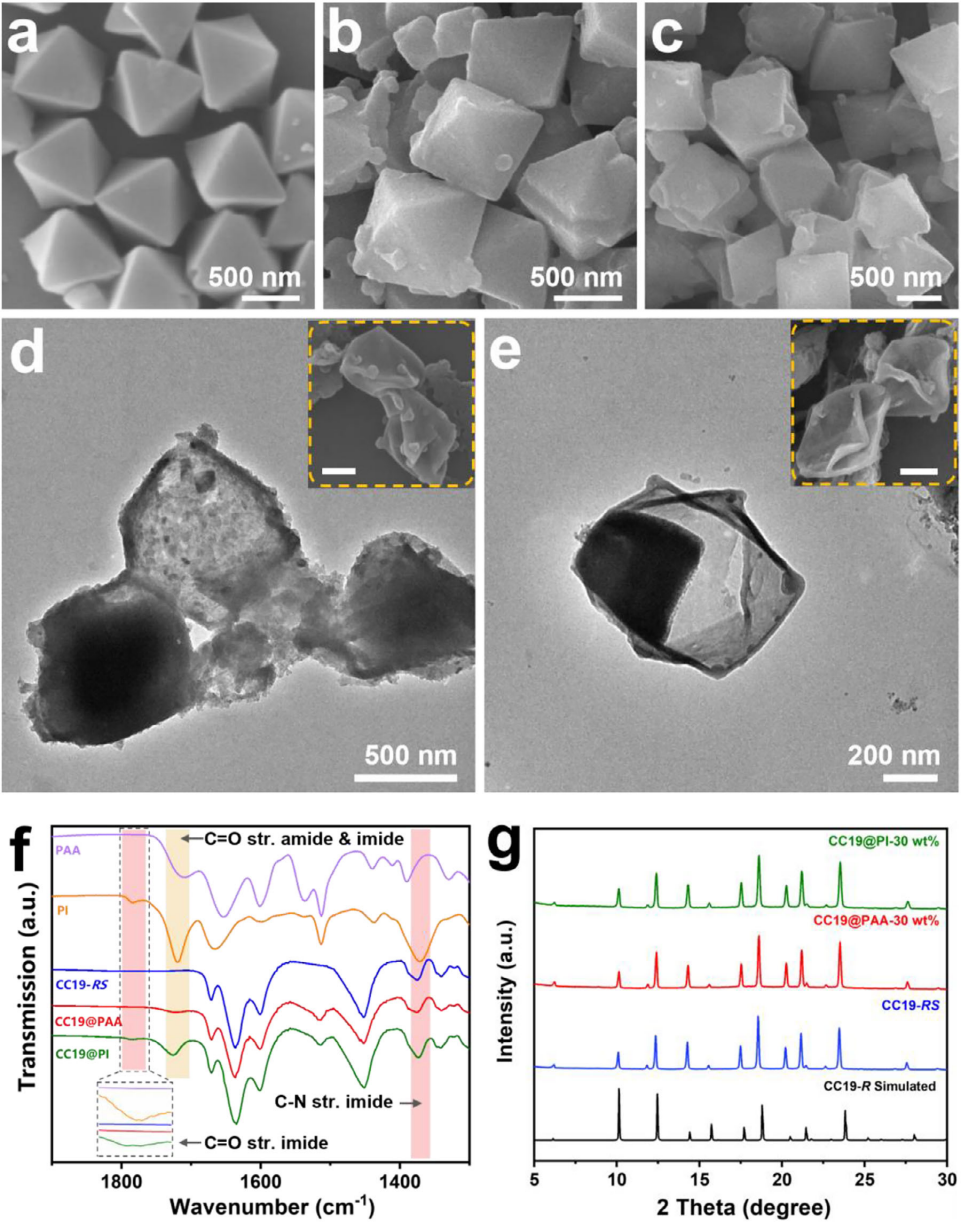
SEM images of CC19‐*RS* (a), CC19@PAA‐30 wt.% (b) and CC19@PI‐30 wt.% (c). The CC19 series employs crystals with a particle size of 700 nm. TEM image and (inset) SEM image of the PAA (d) and PI (e) capsule after cage digestion with a inset scale bar as 500 nm. (f) FT‐IR spectra of neat PAA (purple), neat PI (yellow), CC19‐*RS* (blue), CC19@PAA (red) and CC19@PI (green). (g) PXRD patterns comparison of CC19‐*RS*, CC19@PAA‐30 wt.%, CC19@PI‐30 wt.% with simulated CC19‐*R*.

To evaluate the morphology and structural evolution of the POCs@polymer core–shell structures, we conducted detailed electron microscope and spectroscopic analyses. SEM images clearly show that the pristine CC19‐*RS* particles exhibit a uniform octahedral morphology with smooth, well‐defined surfaces (Figure 2a). In contrast, the surface of the composite particles becomes increasingly textured following polymer coating. The CC19@PAA‐30 wt.% (Figure 2b) and CC19@PI‐30 wt.% (Figure 2c) samples display notably roughened surfaces, consistent with the formation of a polymer shell layer. TEM images further highlight the morphological changes; while uncoated CC19‐*RS* particles show sharp and angular edges, the coated composites exhibit rounded contours indicative of uniform surface coverage and the presence of a soft polymer interface (Figure S9).

To provide direct evidence for the successful formation of a polymer shell and to estimate its thickness, we performed acid etching experiments. CC19‐*RS* cores were selectively digested by immersing the composite particles in 6 M HCl for 6 h at room temperature, taking advantage of the chemical instability of the cage compared to the robust polymer layer. Post‐etching electron microscopy revealed hollow octahedral capsules with preserved external shape, confirming the integrity of the shell structure (Figure 2d,e). The shell thickness was estimated to be ∼20 nm, supporting the conclusion that a conformal and continuous polymer layer was deposited via the NISAP process. To further validate the shell thickness, we performed DLS measurements to compare the particle sizes of uncoated CC19‐*RS* and CC19@PAA. The corresponding shell thickness can therefore be estimated as 22.6 nm, which is in good agreement with the thickness obtained from TEM analysis (Figure S10).

Fourier‐transform infrared (FT‐IR) spectroscopy further validated the chemical composition of the polymer coatings (Figure 2f). The CC19@PAA composite exhibited a characteristic absorption band at 1723 cm^−1^, corresponding to the C═O stretching of the amide group, in agreement with the spectrum of neat PAA. After chemical imidization, the CC19@PI composite displayed two distinct peaks at 1785 and 1385 cm^−1^, which are attributed to the symmetric C═O stretch of the imide group and C─N stretching, respectively. These observations confirm the successful conversion of PAA to polyimide and the preservation of chemical integrity during the shell formation process.

PXRD patterns revealed that both CC19@PAA and CC19@PI retained the crystallographic features of the original CC19‐*RS* material (Figure 2g), closely matching the simulated pattern of the CC19‐*R* crystal structure. This demonstrates that the introduction of an external polymer shell does not perturb the internal packing of the molecular cages or compromise their crystalline packing mode.

Thermal stability was evaluated using thermogravimetric analysis. Both CC19‐*RS* and CC19@polymer composites exhibited similar decomposition temperatures around 375°C, indicating that the polymer shell does not adversely affect the thermal robustness of the cage material, thereby retaining its excellent thermal stability. Furthermore, compositional analysis based on TGA data revealed that the polymer shell accounts for approximately 20.7 wt.% of the total mass in the composite, corresponding to a polymer‐to‐cage mass ratio over 26.1 wt.%. For CC19@PAA, the estimated polymer content is ∼19.0 wt.%, corresponding to a polymer‐to‐cage mass ratio of approximately 23.4 wt.% (Figure S11). Collectively, these results confirm the successful fabrication of CC19@polymer core–shell nanostructures with well‐defined morphology, conformal polymer coating, and preserved crystalline structure, forming a robust platform for further functionalisation and application in molecular separation.

To clarify the applicability of our interfacial synthetic strategy, we extended the same fabrication protocol to another well‐studied imine‐based porous organic cage, CC3 (Figure S12). The resulting CC3@polymer core–shell nanostructures exhibit structural features analogous to those observed for CC19@polymer, including a well‐defined polymer shell and an intact crystalline CC3 core. These results demonstrate that the coating strategy is effective for structurally related imine‐linked POCs. We note that the extension of this approach to POCs with fundamentally different cage chemistries, such as triazine‐, urea‐, or ether‐linked cages, remains to be established and will be the subject of future investigation.

### 2.3 Enhanced Chemical Stability of POCs@Polymer Core–Shell Nanostructures

Although POCs exhibit promising functionality in molecular recognition and separation, their practical deployment remains limited by their intrinsic chemical fragility under harsh conditions. In particular, exposure to strongly acidic environments often leads to rapid structural degradation due to the acid sensitivity of the imine linkages. To assess the protective efficacy of polymer shells against chemical attack, we systematically evaluated the acid resistance of CC19‐*RS*, CC19@PAA, and CC19@PI composites through a sulfuric acid immersion test.

**FIGURE 3:**
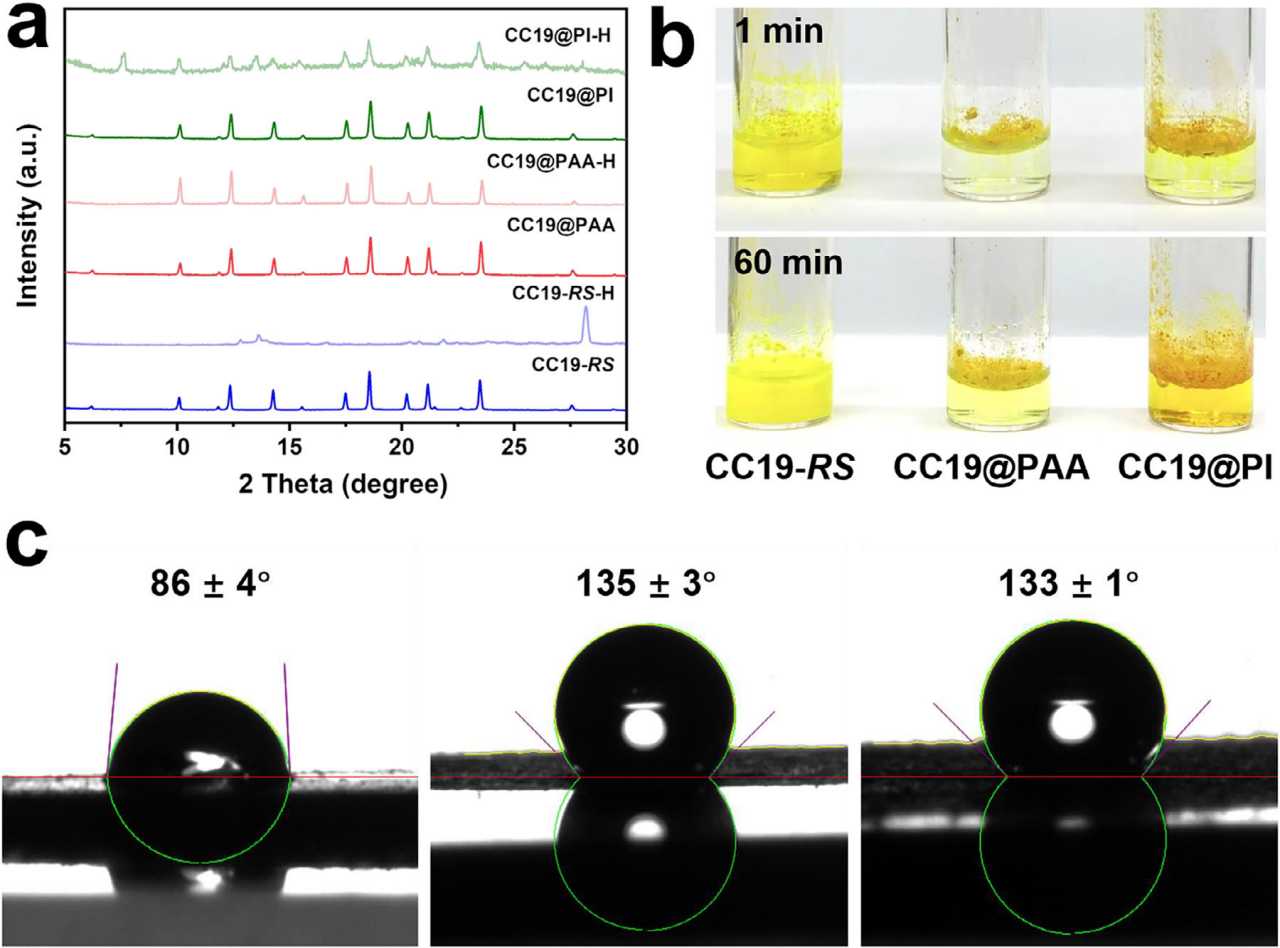
Evaluation of chemical stability and surface wettability of CC19‐based composites. (a) PXRD patterns of CC19‐*RS*, CC19@PAA, and CC19@PI before and after treatment with 6 M H_2_SO_4_ for 60 min. Samples labelled with the suffix “H” (CC19‐*RS*‐H, CC19@PAA‐H, and CC19@PI‐H) were subjected to post‐treatment with 6 M H_2_SO_4_ for 60 min. The sharp diffraction peaks of CC19@PAA are retained post‐treatment, indicating preserved crystallinity, while CC19‐*RS* shows complete loss of crystallinity. (b) Photographs of the dispersions at 1 and 60 min after acid exposure. CC19‐*RS* shows rapid decomposition (yellow coloration), while CC19@PI and CC19@PAA display delayed or minimal colour changes, respectively, consistent with enhanced acid resistance. (c) Water contact angle measurements of CC19‐*RS*, CC19@PAA, and CC19@PI, confirming a transition from hydrophilic to hydrophobic surfaces upon polymer shell formation. The increased hydrophobicity contributes to interfacial acid resistance by minimising proton penetration and promoting phase separation from the acidic medium.

Upon the addition of a sulfuric acid solution to a dispersion of CC19‐*RS*, rapid colouration was observed within 1 min (Figure 3b), indicating immediate cage degradation and dispersion. After 60 min, the system developed a deeper yellow suspension, consistent with extensive chemical decomposition. This observation was corroborated by PXRD, which showed a complete loss of crystallinity in the treated sample (Figure 3a), confirming the structural collapse of the cage network in the absence of polymer protection. In contrast, CC19@PI exhibited only slight yellowing within 1 min, while CC19@PAA showed visually colourless and transparent, indicating significantly delayed degradation. After 60 min, the CC19@PI dispersion turned dark yellow, suggesting partial cage decomposition within the composite. However, the CC19@PAA sample maintained only a faint yellow hue and retained its crystalline diffraction features in PXRD. Together with its well‐retained selective gas adsorption performance after acid treatment (Figure S21), these results indicate that the PAA shell effectively suppresses acid penetration and preserves the structural integrity of the encapsulated cage. As this functionality is highly sensitive to cage degradation, its retention provides a quantitative, function‐based indication that a substantial fraction of the cage remains intact. Fourier transform infrared (FT‐IR) spectroscopy was further employed to compare the materials before and after acid treatment, providing a direct and functional evaluation of the chemical stability of the POCs@polymer core–shell nanostructures relative to uncoated CC19 (Figure S13).

To gain further mechanistic insight into this chemical shielding behavior, we analysed the surface wettability of the three materials via water contact angle measurements (Figure 3c). Pristine CC19‐*RS* exhibited a moderate contact angle of 86 ± 4°, indicative of a hydrophilic surface. In contrast, CC19@PAA and CC19@PI displayed significantly increased contact angles of 135 ± 3° and 133 ± 1°, respectively, reflecting strong hydrophobic character. This transformation is attributed to the incorporation of fluorinated PAA and PI shells, which lowers the surface energy of the material through the presence of perfluorinated groups [32]. The enhanced hydrophobicity contributes to the chemical stability of the composites in multiple ways. First, it reduces the interfacial wettability between the cage particles and the aqueous acid phase, leading to spontaneous interfacial aggregation and the formation of a heterogeneous dispersed system. Second, the low surface energy induced by fluorine‐containing groups effectively inhibits proton diffusion into the cage interior, thus mitigating acid‐catalysed hydrolysis of the imine bonds. This interfacial barrier effect plays a critical role in maintaining the crystalline framework of the POCs under corrosive conditions.

### 2.4 Gas Sorption Behavior and Selectivity Enhancement via Polymer Shell Engineering

**FIGURE 4:**
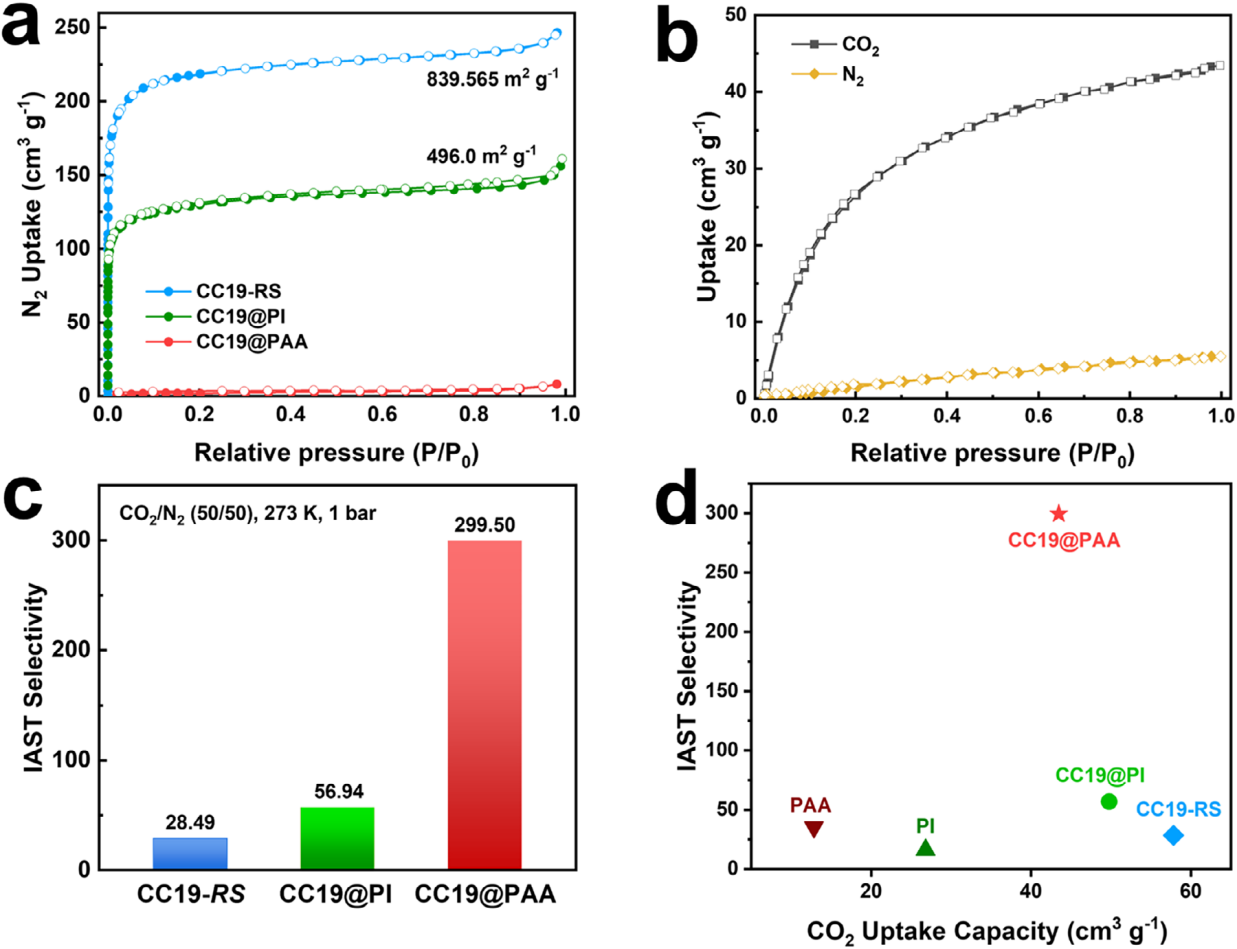
(a) N_2_ adsorption isotherms at 77 K for CC19‐*RS*, CC19@PI, and CC19@PAA, showing reduced N_2_ uptake in polymer‐coated samples, especially in CC19@PAA, indicating partial pore blocking or restricted diffusion. Filled and open symbols represent adsorption and desorption, respectively. (b) CO_2_ and N_2_ adsorption isotherms for CC19@PAA at 273 K for the same series, highlighting enhanced CO_2_ uptake despite its low N_2_ uptake. (c) Ideal Adsorbed Solution Theory (IAST)‐predicted selectivity of CO_2_/N_2_ (50:50, 273 K, 1 bar) for CC19‐*RS*, CC19@PI, and CC19@PAA. (d) The trade‐off between CO_2_ adsorption capacity and CO_2_/N_2_ selectivity was evaluated for PAA, PI, CC19‐*RS*, CC19@PAA, CC19@PI, CC19@PAA demonstrated the optimal overall performance, exhibiting an exceptional balance between CO_2_ adsorption capacity and CO_2_/N_2_ selectivity.

Given the pronounced differences in chemical stability between the PAA and PI shell materials, we hypothesised that the improved stability of POCs@polymer composites may stem from changes in the surface pore architecture, which modulate guest molecule access and transport. To probe this effect, nitrogen (N_2_) sorption measurements were conducted at 77 K to assess microporosity and accessible surface area. As shown in Figure 4a, both CC19‐*RS* and CC19@PI exhibit classical Type I adsorption isotherms, characteristic of microporous materials. The Brunauer–Emmett–Teller (BET) surface areas were determined to be 839.5 m ^2^g^−1^ for CC19‐*RS* m^2^ g^−1^ and 496.0  m ^2^ g^−1^ for CC19@PI, indicating a partial reduction in accessible surface due to the polymer shell. In contrast, CC19@PAA displayed negligible N_2_ uptake, consistent with a nonporous character at cryogenic temperature and suggesting more substantial pore occlusion or restricted gas diffusion. The key origin of the SA discrepancy between CC19@PAA and CC19@PI instead lies in the intrinsic properties of the polymer shells themselves. As shown by N_2_ adsorption isotherms at 77 K (Figure S14), pure PAA exhibits negligible N_2_ uptake (≈0 cm^3^ g^−^^1^ STP), indicating its dense, non‐porous nature toward N_2_ under cryogenic conditions. In contrast, pure PI displays measurable N_2_ adsorption (∼150 cm^3^ g^−^^1^ at P/P_0_ = 1.0), consistent with the presence of accessible porosity.

To gain deeper insight into pore accessibility and size distribution, CO_2_ adsorption isotherms were measured at 273 K, followed by non‐local density functional theory (NLDFT) analysis (Figure S15). The pristine CC19‐*RS* exhibited a CO_2_ uptake of 57.8 cm^3^ g^−1^ at a relative pressure of p/p_0_ at 0.99 and a bimodal pore distribution with peaks at approximately 6.0 Å and 8.2 Å. These values align with crystallographic data [33], corresponding to the window apertures and internal cavity dimensions of the cage structure. Upon polymer encapsulation, CC19@PI retained similar pore features (5.7 Å and 8.2 Å), albeit with slightly diminished uptake, confirming partial pore blocking by the rigid PI layer. Interestingly, CC19@PAA still exhibited a moderate CO_2_ uptake of 43.5 cm^3^ g^−1^, despite its nonporous behavior toward N_2_, and showed a more pronounced reduction in pore size (5.2 Å and 8.2 Å). This suggests that the flexible PAA chains, enriched with polar carboxyl and amide groups, form a denser and more conformal coating layer that narrows the pore entrances, selectively hindering larger or less interactive gas species.

The significant differences in the adsorption behavior of CC19@PAA for CO_2_ and N_2_ prompted us to explore its potential for separating gas mixtures (Figure 4b). As shown, CC19@PAA can take up 5.5 cm^3^ g^−1^ N_2_, which is significantly lower than that of CO_2_ at 273 K, 1 bar. This enhanced selectivity is attributed to two key factors: on the one hand, CO_2_ possess a smaller dynamic diameter of 0.33 nm than N_2_ of 0.36 nm [34], on the other hand, CO_2_ possess a higher polarizability (29.1×10 25 cm^−3^) than N_2_ (17.4×10 25 cm^−3^) [35], this enhanced polarizability promotes preferential interactions between CO_2_ and the polar functional groups within the PAA shell. This mechanism is supported by the significantly higher isosteric heat of adsorption (Q_s__t_) for CO_2_ (32.08 kJ mol^−^^1^) compared with that for N_2_ (9.68 kJ mol^−^^1^). Together, these effects favour CO_2_ adsorption while suppressing N_2_ uptake, thereby enhancing molecular sieving performance. For comparison, the Q_s__t_ values for CO_2_ adsorption on CC19@PI and CC19‐*RS* were calculated to be 31.06 and 23.22 kJ mol^−^^1^, respectively (Figure S16).

Single‐point Langmuir‐Freundlich isothermal models were employed to fit the adsorption isotherm data for pure CO_2_ and N_2_ to quantitatively evaluate the separation potential (Figures S17–S19). The binary selectivity of CO_2_/N_2_ (50:50) mixtures was predicted using Ideal Adsorption Solution Theory (IAST) [36]. At 273 K and 1 bar, the calculated IAST selectivity of CO_2_/N_2_ (50:50) increased from 28.4 to 56.9 for CC19@PI compared to CC19‐*RS*, demonstrating the effectiveness of rigid shell‐induced molecular sieving (Figure 4c; Figure S20). Strikingly, CC19@PAA achieved an exceptional selectivity of 299.5, representing more than a tenfold improvement over CC19‐*RS*. Based on the combined evidence from Q_s__t_ analysis, adsorption behavior, and structural considerations of the core–shell architecture, we propose that the enhanced selectivity of CC19@PAA originates from a synergistic interplay between pore‐size regulation by the dense PAA shell and strengthened polar interactions, rather than from differences in adsorption capacity alone. The PAA coating introduces a constricted and chemically polar interfacial region that preferentially favors CO_2_ through size discrimination and enhanced dipole–quadrupole interactions, while simultaneously limiting N_2_ transport. Furthermore, we compared the separation performance of core–shell materials with that of pure materials. As shown in Figure 4d, CC19@PAA exhibits optimal overall performance, achieving the best balance between CO_2_ adsorption capacity and CO_2_/N_2_ selectivity. This fully validates the unique advantage of core–shell nanostructure design in precisely regulating gas separation performance. Additionally, the selectivity of CC19@PAA at CO_2_/N_2_ = 15:85 under 273 K is 91.4, this level of performance exceeds most reported porous frameworks, including MOFs [37], COFs [38, 39], and porous organic polymers [40] (Table S2).

We performed gas sorption analyses on CC19@PAA before and after acid treatment to demonstrate that the chemical stability of polymer‐coated POCs should be evaluated not only by structural retention but also by the preservation of functional performance (Figure S21). Whereas uncoated CC19 undergoes rapid acid‐induced imine hydrolysis accompanied by a complete loss of adsorption capability, the PAA‐coated core–shell architecture effectively decouples chemical robustness from the intrinsic cage chemistry. The dense, acid‐resistant PAA shell acts as a protective barrier that suppresses acid penetration while maintaining selective gas transport, enabling CC19@PAA to retain both chemical integrity and separation functionality even under harsh chemical conditions. These results underscore the importance of incorporating functional stability metrics—such as post‐treatment adsorption capacity and selectivity—alongside conventional structural characterisation when assessing the durability of porous molecular materials in chemically aggressive environments.

These findings collectively demonstrate that polymer shell engineering, particularly through functional PAA coatings, offers an effective strategy for modulating gas transport and separation behavior in porous organic cage–based materials. The combined effects of size exclusion, polar interactions, and dynamic pore narrowing enable improved selectivity without compromising chemical robustness. We note that adsorption and diffusion kinetics measurements would provide further valuable insight into the relative transport rates of CO_2_ and N_2_ and would allow a more quantitative assessment of mass‐transfer contributions to the separation mechanism. Such studies represent an important direction for future work.

**FIGURE 5:**
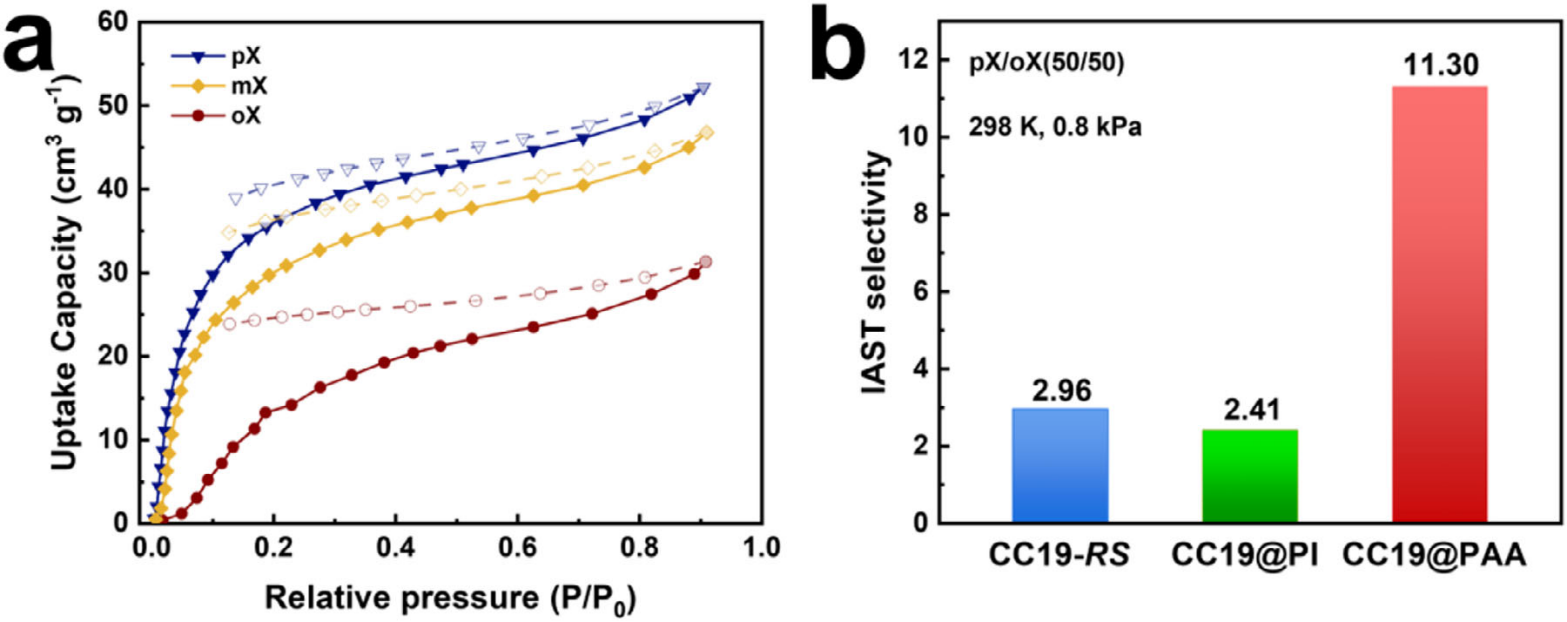
Xylene isomer adsorption and selectivity of CC19@PAA. (a) Adsorption isotherms of PX, MX, and OX on CC19@PAA at 298 K, showing a preferential uptake of PX over its isomeric counterparts. (b) Calculated IAST selectivity for PX over OX (50:50 binary mixture at 298 K, 0.8 kPa) for CC19‐*RS*, CC19@PI, and CC19@PAA, demonstrating an enhancement in separation performance with PAA coating.

Encouraged by the outstanding gas separation performance of CC19@PAA, we further investigated its potential for Xylene isomer separation. As shown in Figure 5a, CC19@PAA exhibited a pronounced uptake preference for PX, adsorbing 52.5 cm^3^ g^−1^ at 298 K and p/p_0_ = 1. In contrast, the uptake of OX under identical conditions was markedly lower at 32.0  cm^3^ g^−1^. This strong sorption selectivity is attributed to the pore‐constraining effect introduced by the PAA shell, which narrows the cage apertures and preferentially accommodates PX due to its more symmetric molecular geometry and smaller effective cross‐sectional area. The larger and less symmetric meta‐Xylene (MX) and OX isomers experience increased steric hindrance, resulting in reduced diffusion and adsorption within the confined pore spaces [41]. To quantify this performance, Ideal Adsorbed Solution Theory (IAST) was applied to predict binary separation efficiencies. At 298 K and 0.8 kPa, the calculated IAST selectivity for PX over OX (50:50 mixture) was 11.30 for CC19@PAA (Figures S22–S24). This represents an enhancement over both CC19‐*RS* (2.96) and CC19@PI (2.41). This fourfold improvement demonstrates that the flexible, polar‐functionalized PAA shell might provide superior molecular recognition through adaptive host‐guest complementarity rather than simple molecular sieving. Our study does not include dynamic breakthrough experiments at this stage. However, fixed‐bed dynamic breakthrough experiments are essential for evaluating the practical separation performance of CC19@PAA under realistic operating conditions, as they provide direct insight into dynamic selectivity, adsorption capacity, and material stability in process‐relevant environments.

These results demonstrate that tailored interfacial polymer coatings not only enhance chemical and thermal robustness but also enable precise molecular discrimination in challenging liquid‐phase separations. The exceptional PX selectivity of CC19@PAA positions this material as a promising candidate for low‐energy, solid‐state separation of industrially relevant xylene isomers, offering a scalable and environmentally benign alternative to traditional thermally driven methods.

## 3 Conclusion

In this study, we have developed a highly efficient interfacial strategy for fabricating POC@Polymer core–shell nanostructures that address fundamental limitations of molecular porous materials while enhancing their performance in separation applications. Our approach uses chiral recognition between enantiomeric cage molecules to produce uniform RS‐type particles, followed by single‐step polymer encapsulation via non‐solvent‐induced surface‐aimed polymerization. This methodology yields conformal polyamic acid and polyimide shells with precisely controlled thickness (∼20 nm) and exceptional monomer utilization efficiency (>90%).

The resulting core–shell architectures successfully combine the intrinsic porosity and synthetic modularity of molecular cages with the mechanical robustness and chemical resistance of high‐performance polymers. Most significantly, the polymer shells provide remarkable protection against chemical degradation, with CC19@PAA maintaining crystalline integrity even after prolonged exposure to concentrated sulfuric acid conditions that rapidly destroy unprotected cage materials. This enhanced stability stems from the hydrophobic character imparted by fluorinated polymer shells, which creates an effective interfacial barrier against proton penetration and acid‐catalyzed hydrolysis.

Beyond stability enhancement, our polymer shell engineering strategy enables unprecedented control over molecular transport and selectivity. The flexible, polar‐functionalized PAA coating narrows pore apertures and introduces favorable dipole‐quadrupole interactions, resulting in exceptional gas separation performance. CC19@PAA achieves a CO_2_/N_2_ selectivity of 299.5, representing a tenfold improvement over pristine cages and exceeding most reported porous materials, including MOFs, COFs, and zeolites under comparable conditions. Additionally, the material demonstrates remarkable xylene isomer discrimination, with PX / OX selectivity enhanced fourfold compared to uncoated cages.

These findings establish polymer shell engineering as a powerful strategy for transforming inherently fragile molecular cages into robust, high‐performance separation materials. The synergistic combination of size exclusion, chemical functionality, and dynamic pore modulation provides a versatile platform for addressing critical challenges in gas separation, chemical purification, and selective molecular transport. The performance characteristics demonstrated herein, including enhanced chemical stability, dramatically improved selectivity, and retained processability‐position POC@Polymer core–shell nanostructures as promising candidates for practical deployment in demanding separation applications, from carbon capture and purification to petrochemical processing and environmental remediation.

## Conflicts of Interest

The authors declare no conflicts of interest.

## Supporting information




**Supporting File**: advs73954‐sup‐0001‐SuppMat.docx.

## Data Availability

The data that support the findings of this study are available from the corresponding author upon reasonable request.
