# Supplementary material for: Enhancing Chemical Stability and Molecular Selectivity of Porous Organic Cages via Core–Shell Polymer Coating
Source: Adv Sci (Weinh). 2026 Jan 27;13(18):e21917. doi: 10.1002/advs.202521917 (PMC13042925; doi:10.1002/advs.202521917)
Supplement: Supplementary file 1 — Supporting File: advs73954‐sup‐0001‐SuppMat.docx. [file ADVS-13-e21917-s001.docx]

**Supporting Information**

**Enhancing Chemical Stability and Molecular Selectivity of Porous Organic Cages via Core-Shell Polymer Coating**

Danyu Li, ^[a]^ Yanling Huang, ^[a]^ Huiyu Liu, ^[a]^ Yuzhen Wen, ^[a]^ Dongxu Wang, ^[a]^ Tao Li*^[b]^ and Shan Jiang*^[a]^

[a] School of Physical Science and Technology, ShanghaiTech University, Shanghai 201210, China

[b] School of Physics, Chemistry and Earth Sciences, University of Adelaide, Adelaide, SA 5005, Australia

**Table of Contents**

**Section S1.** Materials **2**

**Section S2.** Synthetic Details **2**

**Section S3.** Characterization Methods **3**

**Section S4.** Characterizations of POCs **5**

**Section S5.** Characterizations of POCs@polymer Core-Shell Nanostructures **8**

**Section S6.** Gas Sorption Behaviour of POCs@polymer Core-Shell Nanostructures **13**

**Section S7.** Vapor Sorption Behaviour of POCs@polymer Core-Shell Nanostructures **17**

**Section S8.** Vapor Sorption Behaviour of POCs@polymer Core-Shell Nanostructures **18**

**References. 19**

**Section S1. Materials**

(R,R)-1,2-Diaminocyclohexane (R,R-CHDA), (S,S)-1,2-diaminocyclohexane (S,S-CHDA), 1,3,5-triformylbenzene, 2-hydroxy-1,3,5-triformylbenzene, 4,4'-(hexafluoroisopropylidene)diphthalic anhydride (6FDA), and 1,3,5-tris(4-aminophenyl)benzene (TAPB) were purchased from commercial sources and used without further purification. All solvents were obtained from commercial suppliers and used as received.

**Section S2. Synthetic Details**

**2.1 Synthesis of porous organic cages**

**CC19-*R* or CC19-*S* synthesis.** CC19-*R* or CC19-*S* was prepared using a modified literature procedure.^[1]^ *R,R*-CHDA (540 mg, 4.73 mmol) was dissolved in methanol (60 mL) and added dropwise to a solution of 2-hydroxy-1,3,5-triformylbenzene (550 mg, 3.09 mmol) in dichloromethane (250 mL). The reaction mixture was stirred at room temperature for 3 days. The solvent was removed under reduced pressure, and the resulting solid was washed with a methanol/dichloromethane mixture (95:5, v/v) to yield CC19-*R* as a yellow powder (yield: 62.3%).

The synthesis of CC19-*S* was identical to that of CC19-*R*, apart from the use of the *S,S*-CHDA. ^1^H NMR (400 MHz, CDCl_3_) δ 8.69 – 8.53 (m, CH=N, 4H), 8.34 – 8.23 (m, CH=N,4H), 8.08 (m, CH=N,4H), 7.89 (dt, ArH, 8H), 3.32 (d, CH–N, 12H), 1.9 – 1.4 (m, CH_2_, 48H) ppm.

**CC19-*RS* racemic particles.** Racemic cage nanoparticles were prepared by controlled mixing of enantiomeric solutions. The procedure is adapted for a previous study.^[2]^ CC19-*R* (1.5 mg mL⁻¹ in dichloromethane, 7 mL) and CC19-*S* (1.5 mg mL⁻¹ in dichloromethane, 7 mL) solutions were combined using a syringe pump at 0.2 mL min⁻¹ under ambient conditions The mixed the solution was left over 2 h to form the racemic crystals. The resulting yellow precipitate was collected by filtration through a 450 nm nylon membrane.

**2.2 Core-shell nanostructure fabrication**

**POCs@PAA synthesis.** CC19-*RS* particles (10 mg) were dispersed in 2 mL N,N-dimethylformamide (DMF) by sonication, collected by centrifugation, and redispersed in 4 ml dichloromethane (DCM). Solutions of 780 μl 6FDA (5 mg in 2 ml DCM) and 24 μl TAPB (5 mg in 117.6 μl DMF) were added to achieve a calculated polymer loading of 30 wt%. Petroleum ether (20 mL) was rapidly added under stirring (600 rpm) to induce phase separation. After 30 min, 20 ml methanol was added to quench the reaction, and stirring continued for an additional 30 min. The CC19@PAA product was collected by centrifugation and washed three times with petroleum ether.

**POCs@PI synthesis.** Chemical imidization was performed to convert PAA to polyimide. 30 mg CC19@PAA was dispersed in 30 ml DMF, followed by addition of 6.34 ml acetic anhydride and 2.36 ml triethylamine. The mixture was stirred for 4 h at room temperature. The CC19@PI product was collected by filtration and washed with DMF and DCM.

**2.3 Chemical stability assessment.**

The acid resistance of core-shell nanostructures was evaluated through controlled exposure to sulfuric acid. Samples (10 mg) were immersed in 1 M H_2_SO_4_ (1 mL) at room temperature to stand for 1h. Following acid treatment, materials were collected by vacuum filtration through a 0.45 μm PTFE membrane, washed thoroughly with deionized water until neutral pH was achieved, and dried under vacuum at 40°C for 12 h. Structural integrity was assessed by comparing PXRD patterns before and after acid exposure. Visual changes in sample morphology and coloration were documented photographically at regular intervals during the treatment period.

**2.4 Selective core etching experiments.**

To demonstrate hollow capsule formation and estimate shell thickness, cage cores were selectively dissolved while preserving the polymer shell. Core-shell samples (10 mg) were dispersed in 6 M HCl (1 mL) and maintained at room temperature for 6 h with periodic gentle agitation. The heterogeneous mixture was then filtered through a 0.22 μm PTFE membrane to collect the insoluble polymer shells. The recovered material was washed sequentially with deionized water (3 × 10 mL) to remove residual acid and cage decomposition products. Finally, the purified hollow capsules were redispersed in methanol for electron microscopy analysis.

**Section S3. Characterization Methods**

**Electron microscopy.** Scanning electron microscopy (SEM) images were acquired using a JSM-IT500HR/LA field-emission scanning electron microscope. Transmission electron microscopy (TEM) images were obtained on a JEM-1400 field-emission transmission electron microscope operating at 120 kV. Samples were prepared by drop-casting dilute suspensions onto carbon-coated copper grids and allowing the solvent to evaporate under 40 °C.

**Spectroscopic analysis.** Fourier-transform infrared (FT-IR) spectra were recorded on a Thermo Scientific Nicolet iS50 spectrometer over the range 400–4000 cm⁻¹ with 4 cm⁻¹ resolution. ¹H NMR spectra were acquired on a Bruker AVANCE III HD 400 MHz spectrometer at room temperature using CDCl₃ as solvent and tetramethylsilane as internal standard.

**Powder X-ray diffraction.** Powder X-ray diffraction (PXRD) patterns were recorded on a Bruker D8 Advance diffractometer using Cu Kα radiation (λ = 1.5418 Å). The scan rate was 1 s per step with a step size of 0.02^o^.

**Thermal analysis.** Thermogravimetric analysis (TGA) was performed using a PerkinElmer TGA 8000 and Thermo Fisher ISQ 7610 thermal analyzer under nitrogen atmosphere. Samples were initially heated to 30°C and maintained at this temperature for 10 min, followed by heating to 700°C at a rate of 5°C min⁻¹ under continuous nitrogen flow (20 mL min⁻¹).

**Contact angle measurements.** Water contact angles were measured using a SL200KS drop shape analyzer. Deionized water droplets (2 μL) were deposited onto sample surfaces, and contact angles were determined using the sessile drop method with at least three measurements per sample.

**Gas adsorption analysis.** Nitrogen adsorption-desorption isotherms were measured at 77 K using a Multi-Port gas physisorption apparatus BELSORP-MAX II volumetric gas adsorption analyzer. Carbon dioxide and nitrogen single-component isotherms were collected at 273 K. Samples were degassed at 100°C under vacuum for 12 h prior to measurements. Brunauer-Emmett-Teller (BET) surface areas were calculated from nitrogen isotherms in the relative pressure range 0.01–0.30. Pore size distributions were determined using non-local density functional theory (NLDFT) analysis applied to CO_2_ adsorption data.

**Vapor adsorption analysis.**

**Selectivity calculations.** Ideal Adsorbed Solution Theory (IAST) was employed to predict binary gas separation selectivities.^[3]^ Single-component isotherms were fitted using Single-site Langmuir-Freundlich models (SLF), and selectivity factors were calculated for equimolar gas mixtures at specified conditions.

Adsorption isotherm of CO_2_, N_2_ at 273 K, and p-Xylene (PX), o-Xylene (OX), at 298 K was fitted by SLF equation

For Single-site Langmuir-Freundlich equation:

$$N=A_{1}\frac{b_{1}P^{c_{1}}}{1+b_{1}P^{c_{1}}}$$

$N$: Molar loading of species (mmol/g)

$A_{1}$: Saturation capacity of species (mmol/g)

$b_{1}$: Constant (Pa^-1^)

$c_{1}$: Constant

$P$: the pressure of the bulk gas at equilibrium with the adsorbed phase (kPa)

IAST selectivity of binary gas mixtures was predicted as follow:

S$=\frac{x_{1}}{x_{2}}\times\frac{y_{2}}{y_{1}}$

$x_{i}$: adsorption amount (mmol/g)

$y_{i}$: mole fractions in the gas phase for the mixtures

The calculated IAST adsorption selectivity for the CO_2_/ N_2_ (CO_2_/ N_2_ = 1/1; v/v) and PX/ OX (PX/ OX = 1/1; v/v) mixtures taking the mole fractions $y_{1}$ = 0.5 and $y_{2}$ = 1-$y_{1}$ = 0.5 for a total pressure of 101 kPa at 273 K and 0.8 kPa at 298K, respectively.

**Isosteric heats (Q_st_) calculations.** The adsorption isotherms of N_2_,CO_2_ were performed on a BELSORP-max II at 273 K,283 K 298 K Q_st_ of CC19-*RS*, CC19@PAA and CC19@PI for CO_2_, N_2_were calculated from adsorption isotherm at 273 K, 283 Kand 298 K by the virial method:

$$\ln P=\ln N+\frac{1}{T}\sum_{i=0}^{n} a_{i}N^{i}+\sum_{i=0}^{m} b_{i}N^{i}$$

P: Pressure (Pa)

N: Uptake (mmol/g)

T: Temperature (K)

a_i_, b_i_: virial coefficients

n, m: Number of coefficients required to adequately express isotherms.

R: universal gas constant (8.314 J mol^–1^ K^–1^)

$$Q_{st}=-\frac{R}{1000}\sum_{i=0}^{n} a_{i}N^{i}$$

**Section S4. Characterizations of POCs**


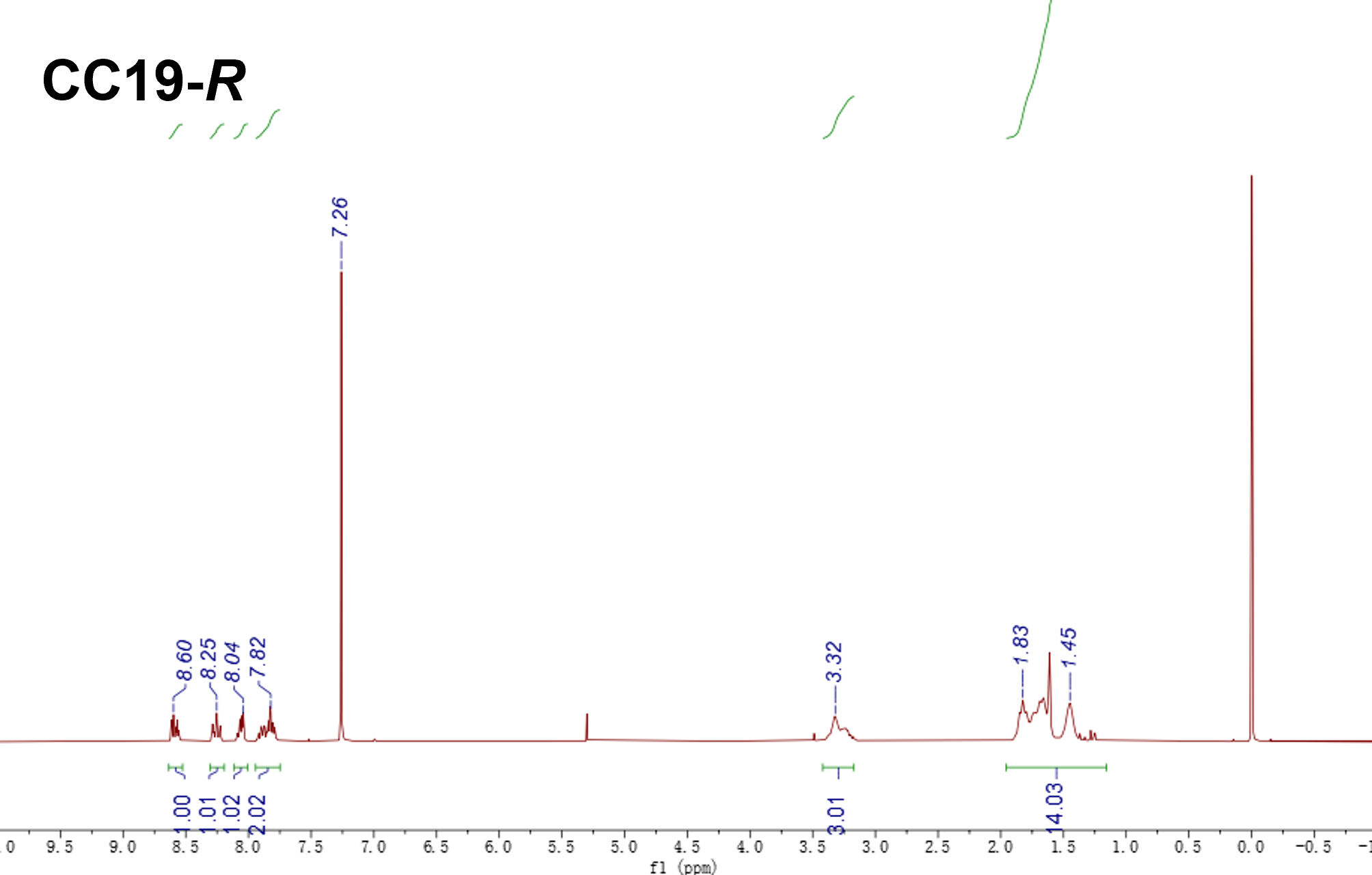


**Figure S1.** ^1^H NMR spectrum (CDCl_3_) of CC19-*R*.


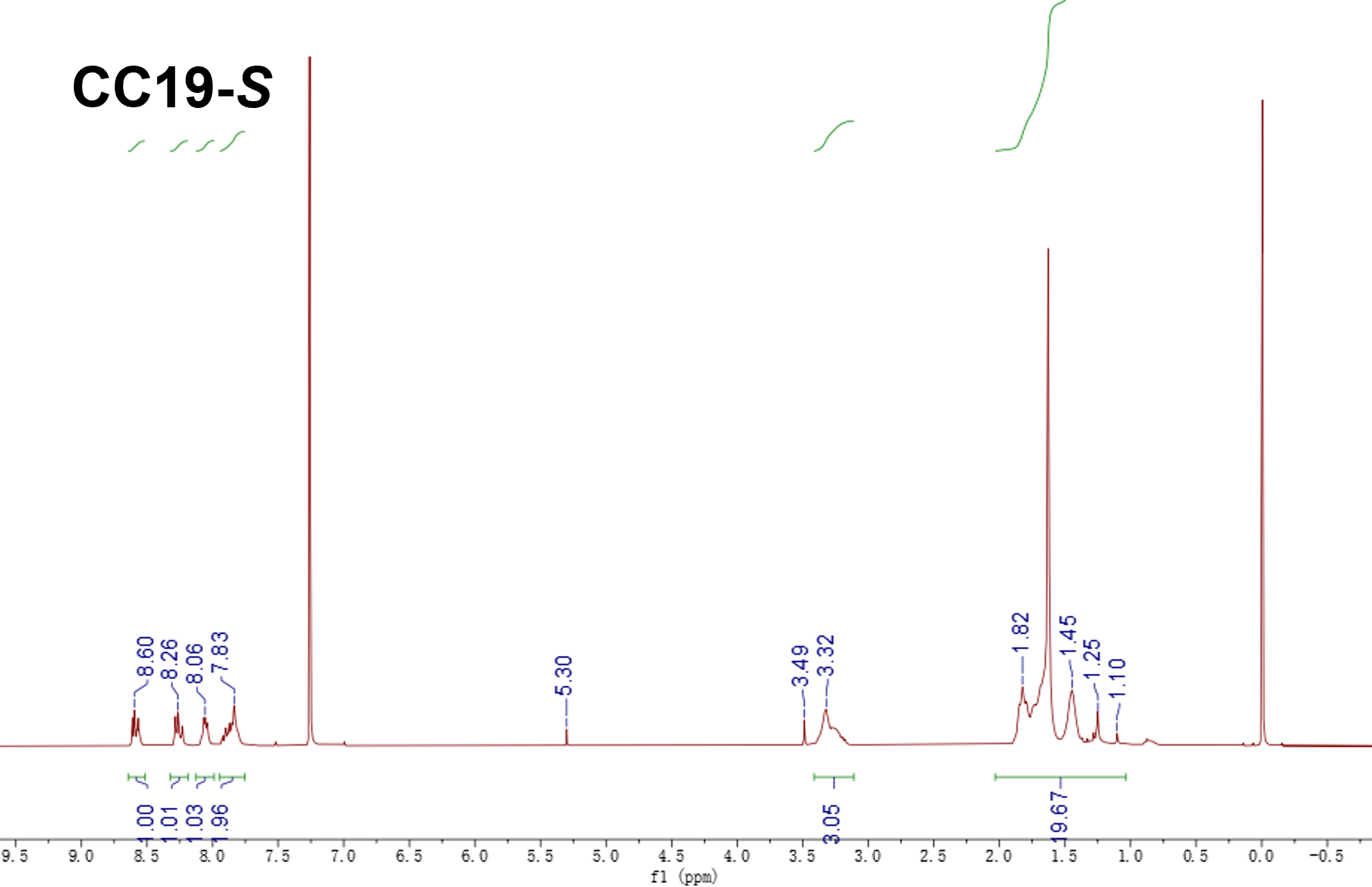


**Figure S2.** ^1^H NMR spectrum (CDCl_3_) of CC19-*S*.

**Table S1**. Controlled synthesis of size-tunable racemic CC19-*RS* particles. Racemic CC19-*RS* cage particles with uniform octahedral morphologies were synthesized through controlled mixing of enantiomeric cage solutions. The particle size was systematically tuned from 350 nm to 1.2 μm by controlling mixing temperature and reaction time parameters. The mixing temperature and subsequent aging time were varied to achieve different particle sizes: 350 nm particles: Mixing at -10°C followed by 10 min aging time; 700 nm particles: Mixing at 0°C followed by 10 min aging time. 1.2 μm particles: Mixing at 20°C followed by 1 h aging time.

| CC19-*RS* particle size / nm | Solvent | C / mg/ml | Volume / ml | T / ℃ | Addition rate (ml/min) | Reaction time (min) |
| --- | --- | --- | --- | --- | --- | --- |
| 1600 | DCM | 1.5 | 8 | 40 | 0.2 | 60 |
| 1200 | DCM | 1.5 | 8 | 20 | 0.2 | 60 |
| 700 | DCM | 2 | 5 | -10 | 2 | 10 |
| 350 | DCM | 1.5 | 5 | -30 | 2 | 10 |


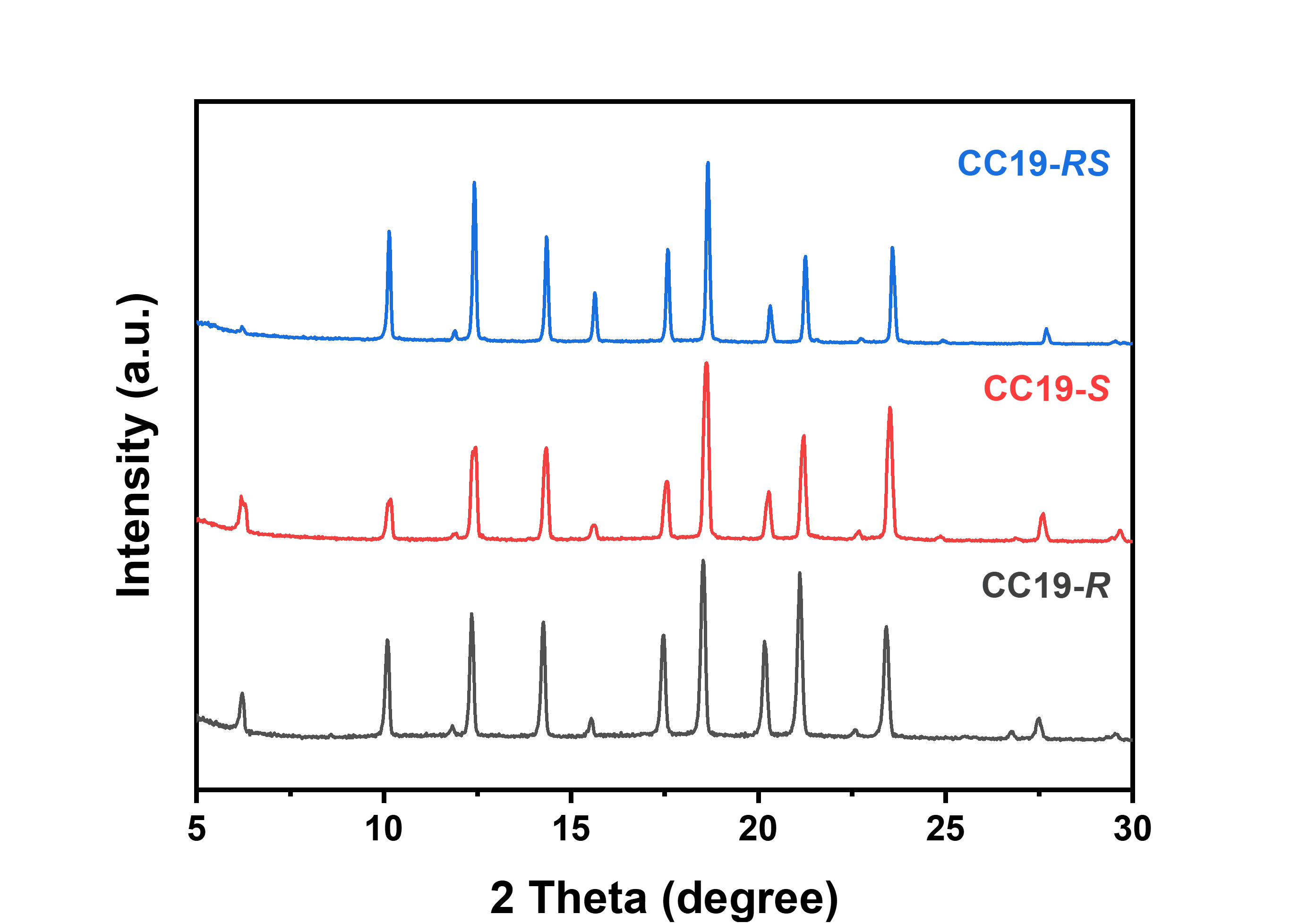


**Figure S3.** PXRD patterns of racemic crystals CC19-*RS* and monochiral crystal CC19-*R* / CC19-*S*. PXRD analaysis confirmed that the racemic cages adopt a crystal packing arrangement identical to that of the enantiopure counterparts.


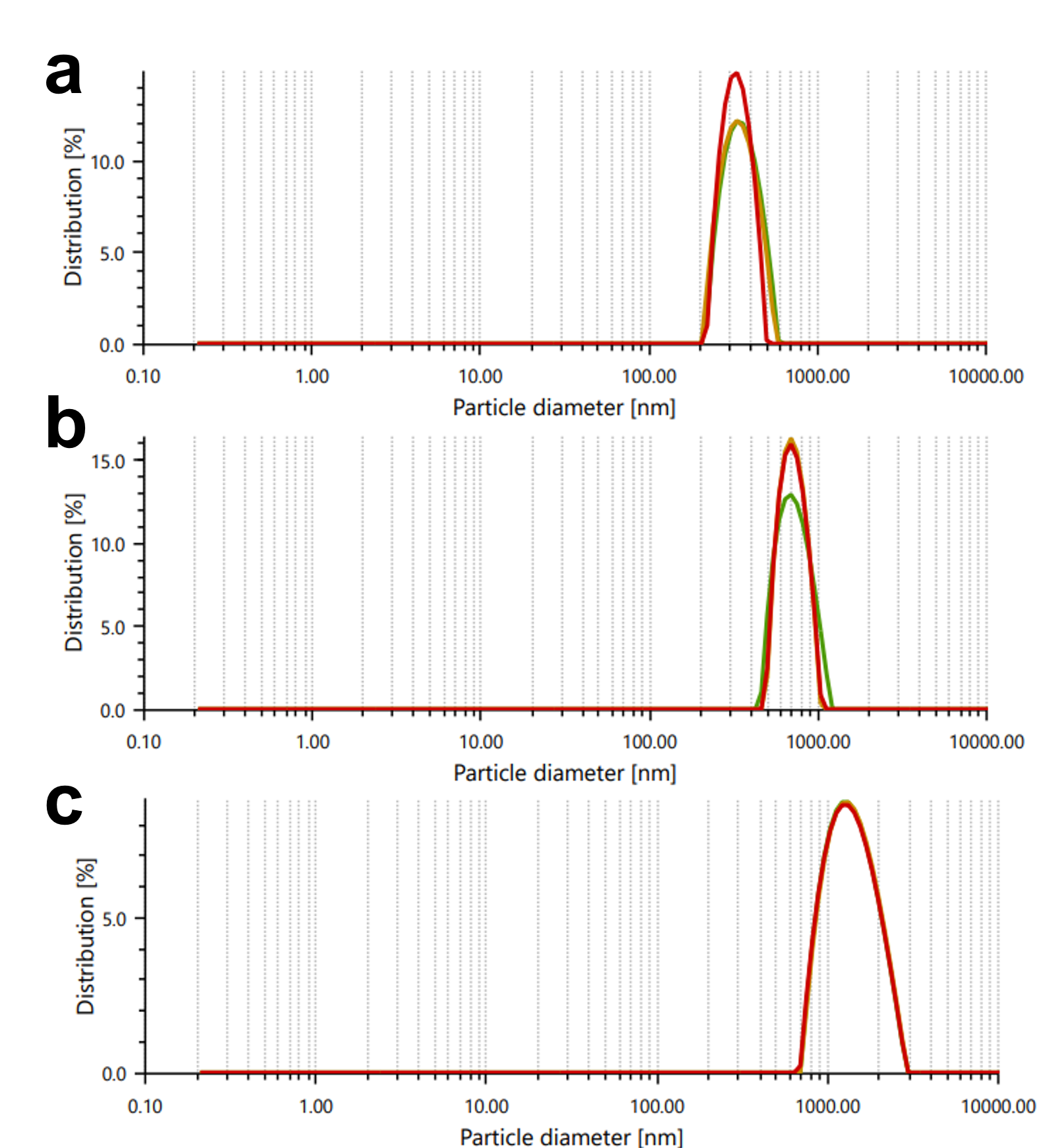


**Figure S4.** Dynamic light scattering (DLS) measurements were conducted in triplicate to determine the particle size distributions of CC19-*RS* samples with different sizes. The average particle sizes and corresponding polydispersity indices (PDI) were: (a) 347 nm (PDI = 0.08), (b) 717 nm (PDI = 0.33), and (c) 1236 nm (PDI = 0.23).


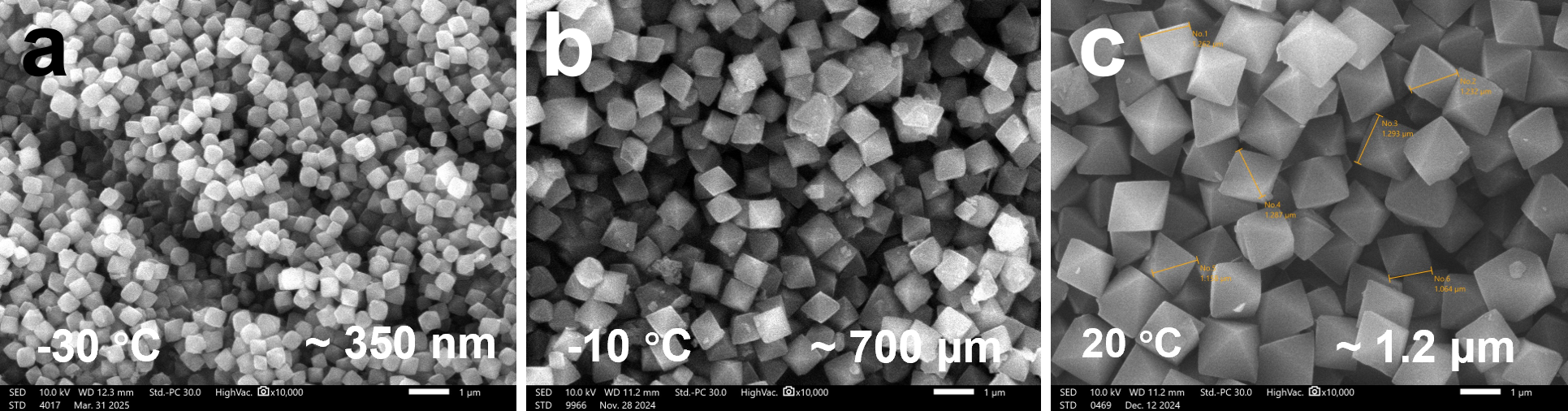


**Figure S5.** SEM images of CC19-*RS* particles synthesised at different temperatures demonstrate a uniform tetrahedral morphology, with particle sizes of (a) 350 nm, (b) 700 nm, (c) 1.2 μm.

**Section S5. Characterizations of POCs@polymer Core-Shell Nanostructures**


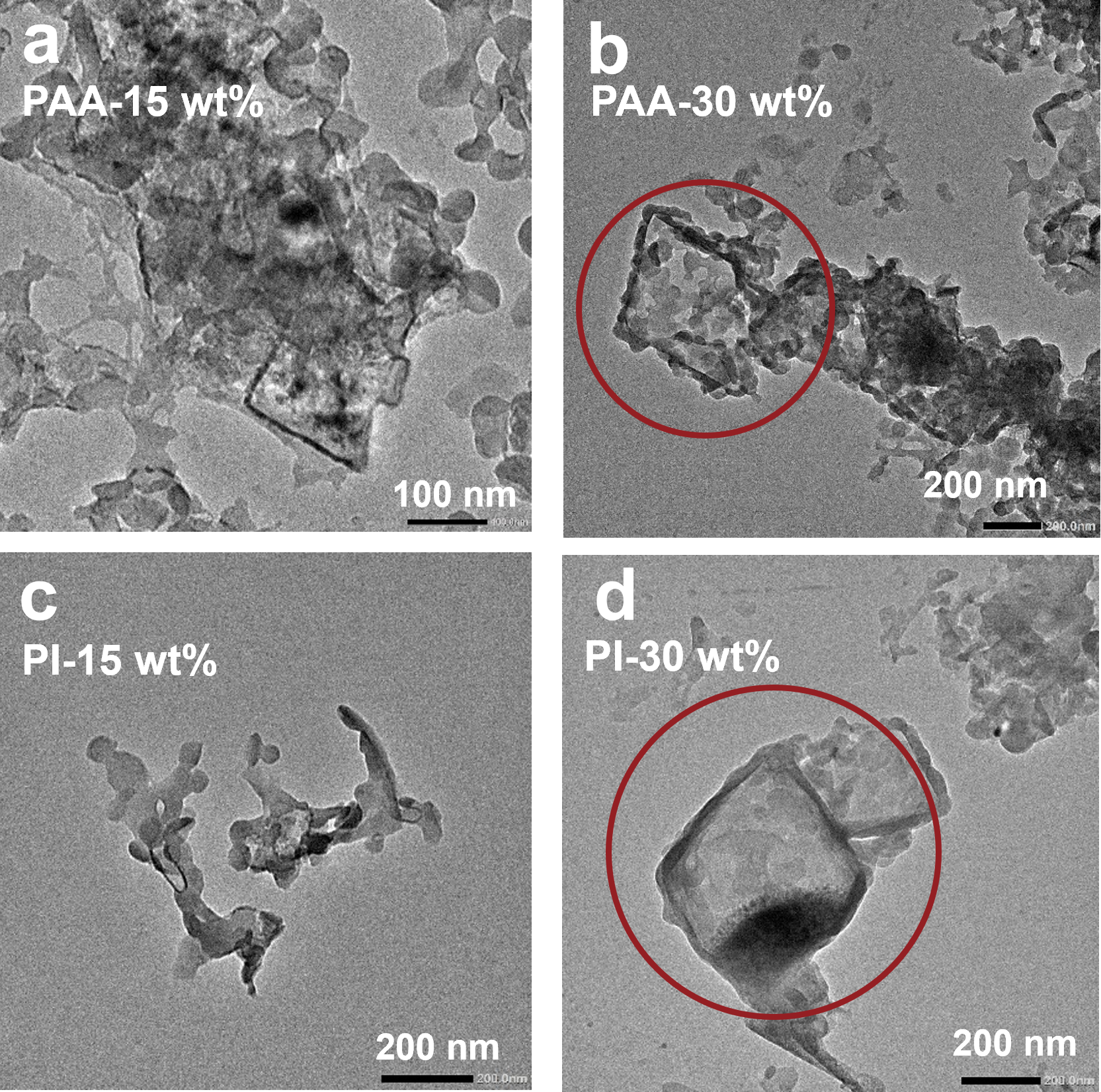


**Figure S6.** TEM images of PAA (a) 15 wt% (b) 30 wt% and PI (c) 15 wt% (dd) 30 wt% capsules, prepared with varying polymer loading ratios and subsequently etched, indicate that at a loading of 15 wt%, the polymer fails to form a continuous coating on the cage surface. Following 6 hours of etching with 6 M hydrochloric acid, the chemically unstable cage core was fully decomposed, while the polymer shell remained intact in samples with a 30 wt% polymer loading. These results confirm that complete surface coverage is achieved at 30 wt%, whereas 15 wt% leads to only partial encapsulation.


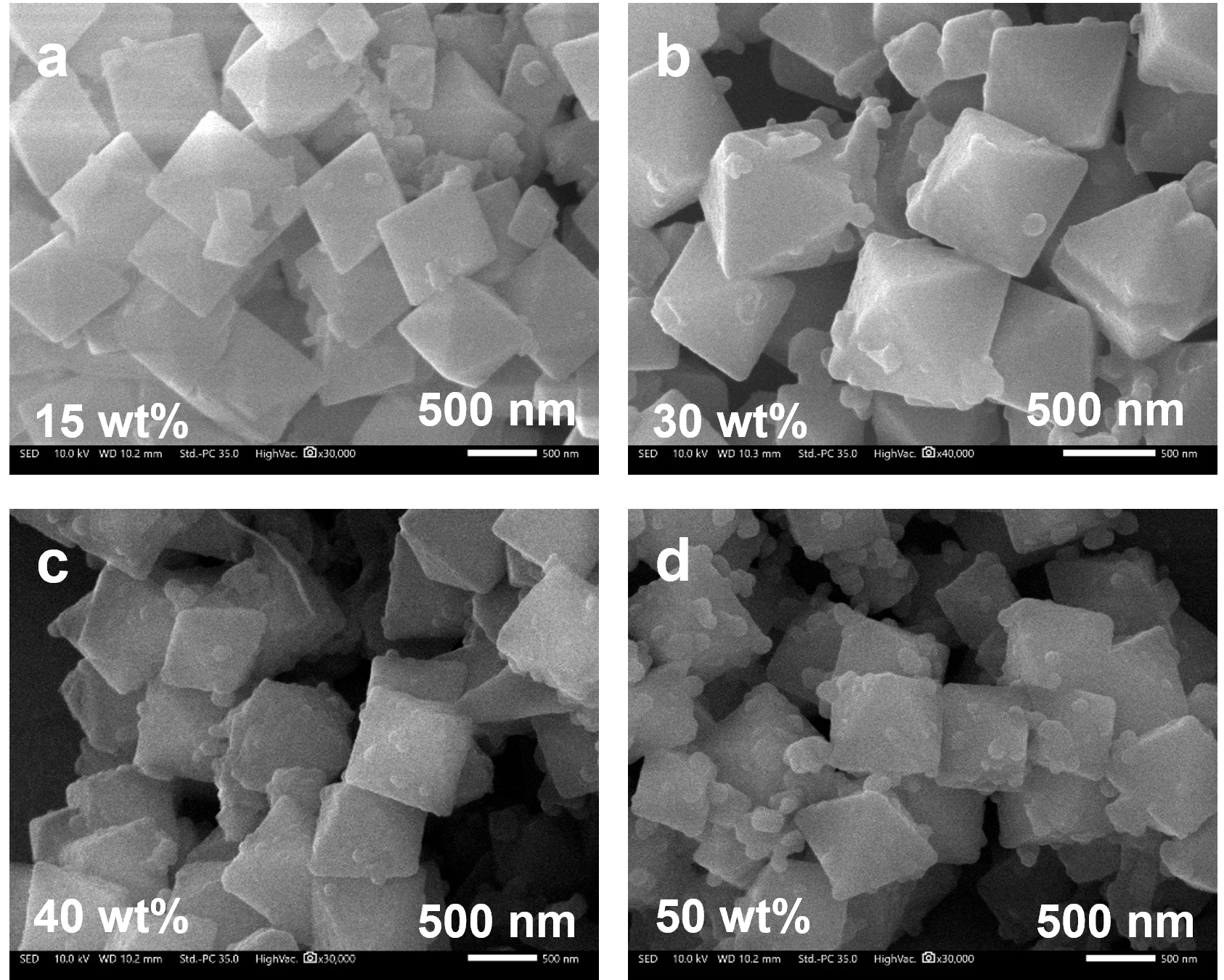


**Figure S7**. SEM images of CC19@PAA with different polymer loading ratios (a) 15 wt%, (b)30 wt%, (c)40 wt%, and (d) 50 wt% show that higher loadings (40-50 wt%) lead to excessive oligomer formation, which compromises the uniformity of the polymer coating.


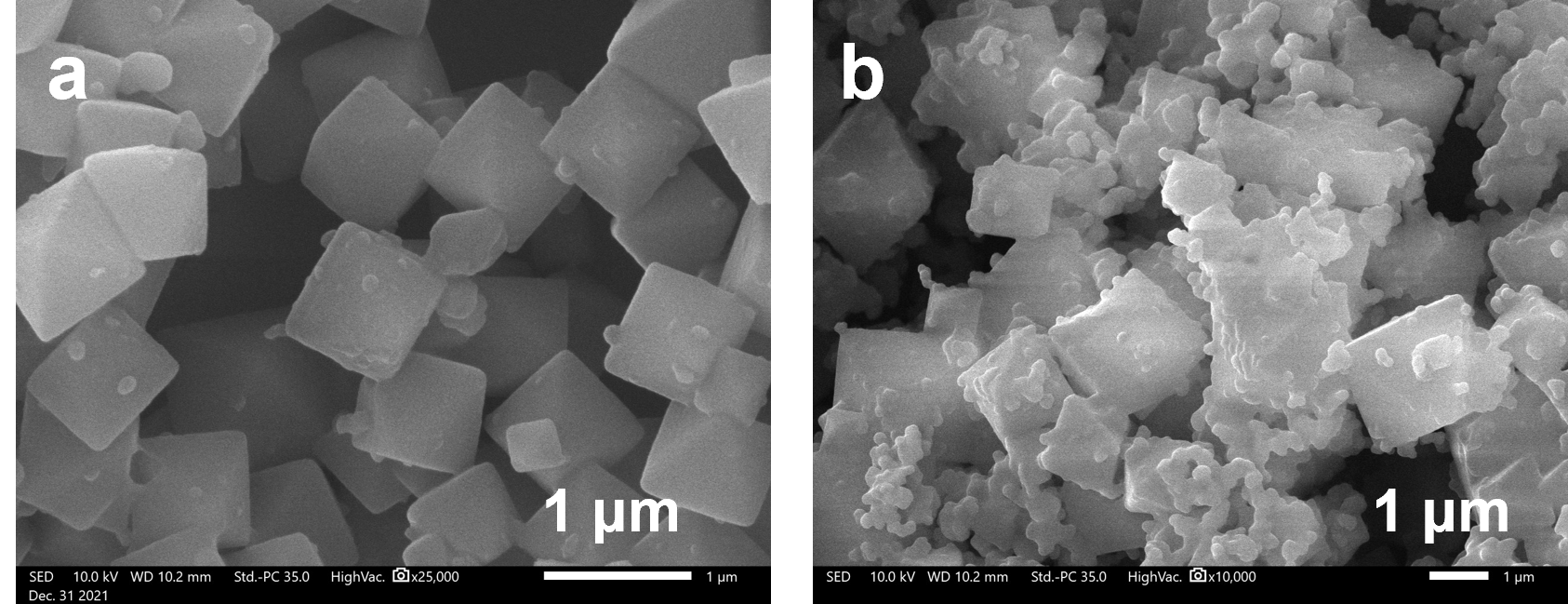


**Figure S8.** SEM images of CC19@PAA at a fixed polymer loading of 50 wt% show that as the cage particle size increases from 700 nm to 2 μm, the amount of surface-bound oligomers increases significantly, particularly when the particle size reaches 2 μm.


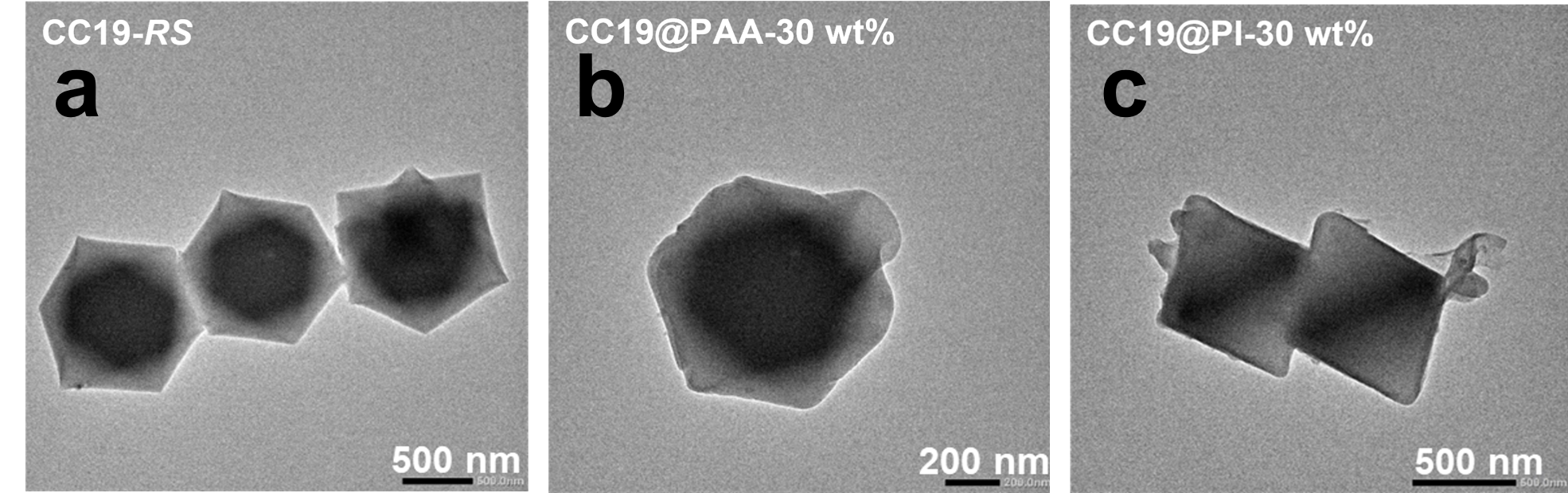


**Figure S9.** TEM images of CC19-*RS* (a), CC19@PAA-30wt% (b), CC19@PI-30wt% (c).


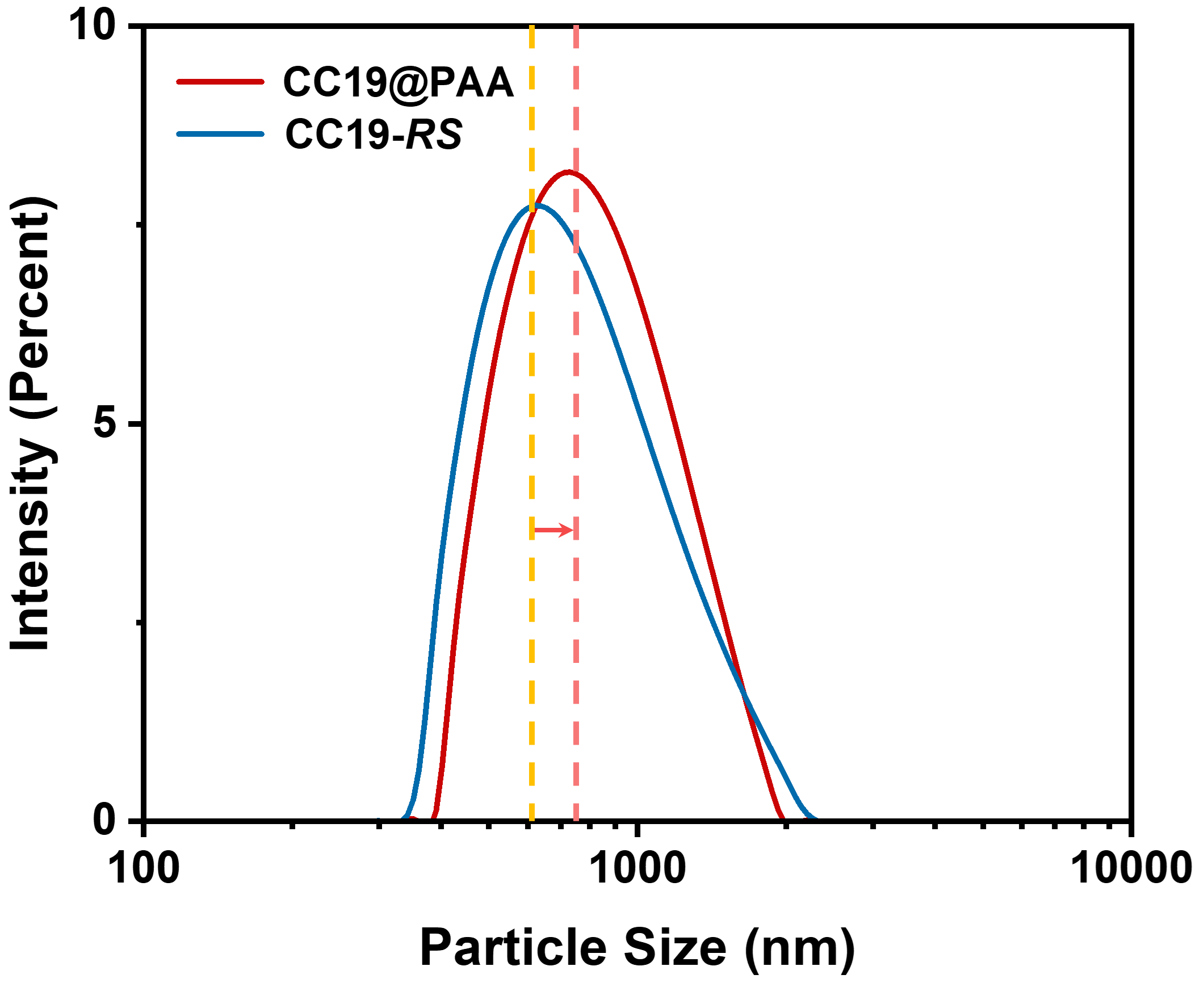


**Figure S10.** Hydrodynamic particle size of CC19-*RS* and CC19@PAA in DCM corresponding to before and after surface modification.


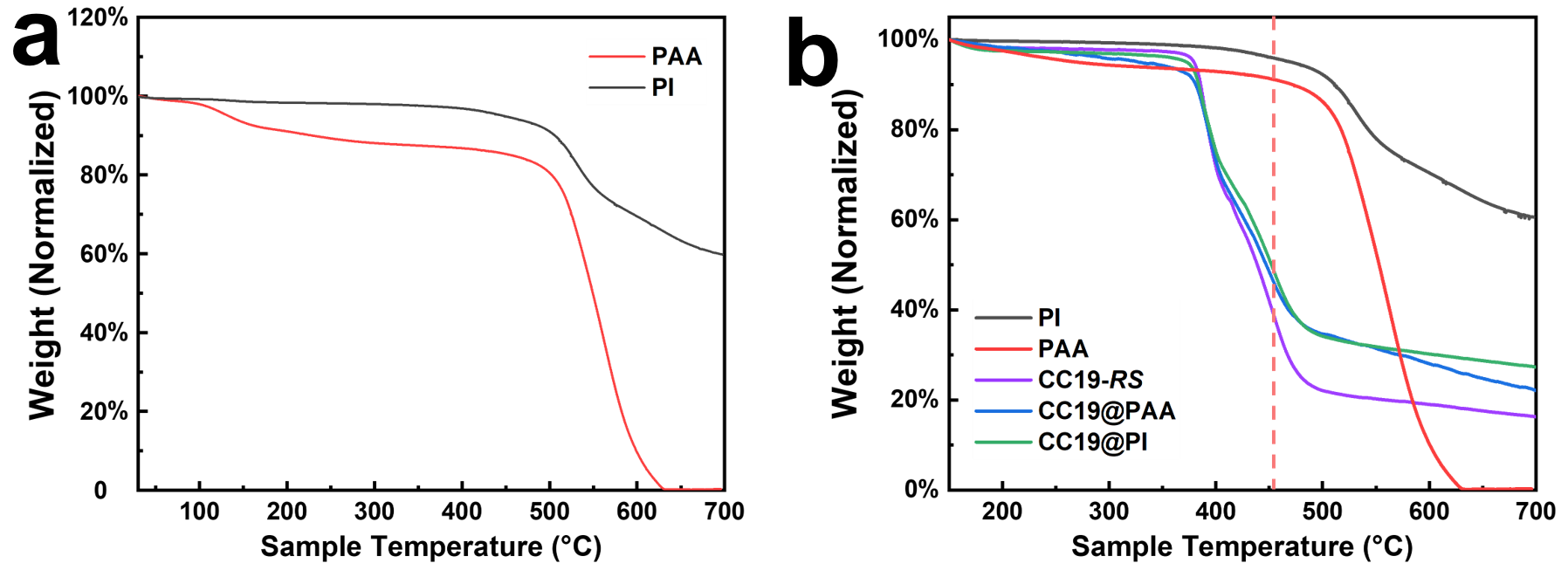


**Figure S11.** (a) TGA curves of PAA and PI under N_2_ atmosphere. (b) TGA curves of CC19-*RS*, CC19@PAA and CC19@PI under N_2_ atmosphere. As shown, The weight of CC19@polymer is 48.21%, showed a significant gap with the 38.25% of CC19-*RS* at 455°C, revealing the POCs@polymer composite with polymer loading: (1-38.25%/48.21%)*100%=20.7%, corresponding to a polymer-to-cage mass ratio of ~26.1 wt%. Furthermore, CC19@PI was synthesized through chemical imidization of CC19@PAA. This conversion involves dehydration-cyclization of the PAA shell, resulting in a reduction of the shell mass. It can therefore be inferred that the actual initial polymer loading in CC19@PAA is higher than the value of 26.1 wt% in CC19@PI. This finding further confirms the excellent monomer utilization efficiency of this system. The polymer loading in CC19@PAA was estimated using the same approach. At 455 °C, CC19@PAA exhibits a residual mass of 47.2%, yielding an estimated PAA content of ~19.0 wt%, which corresponds to a polymer-to-cage mass ratio of approximately 23.4 wt%.


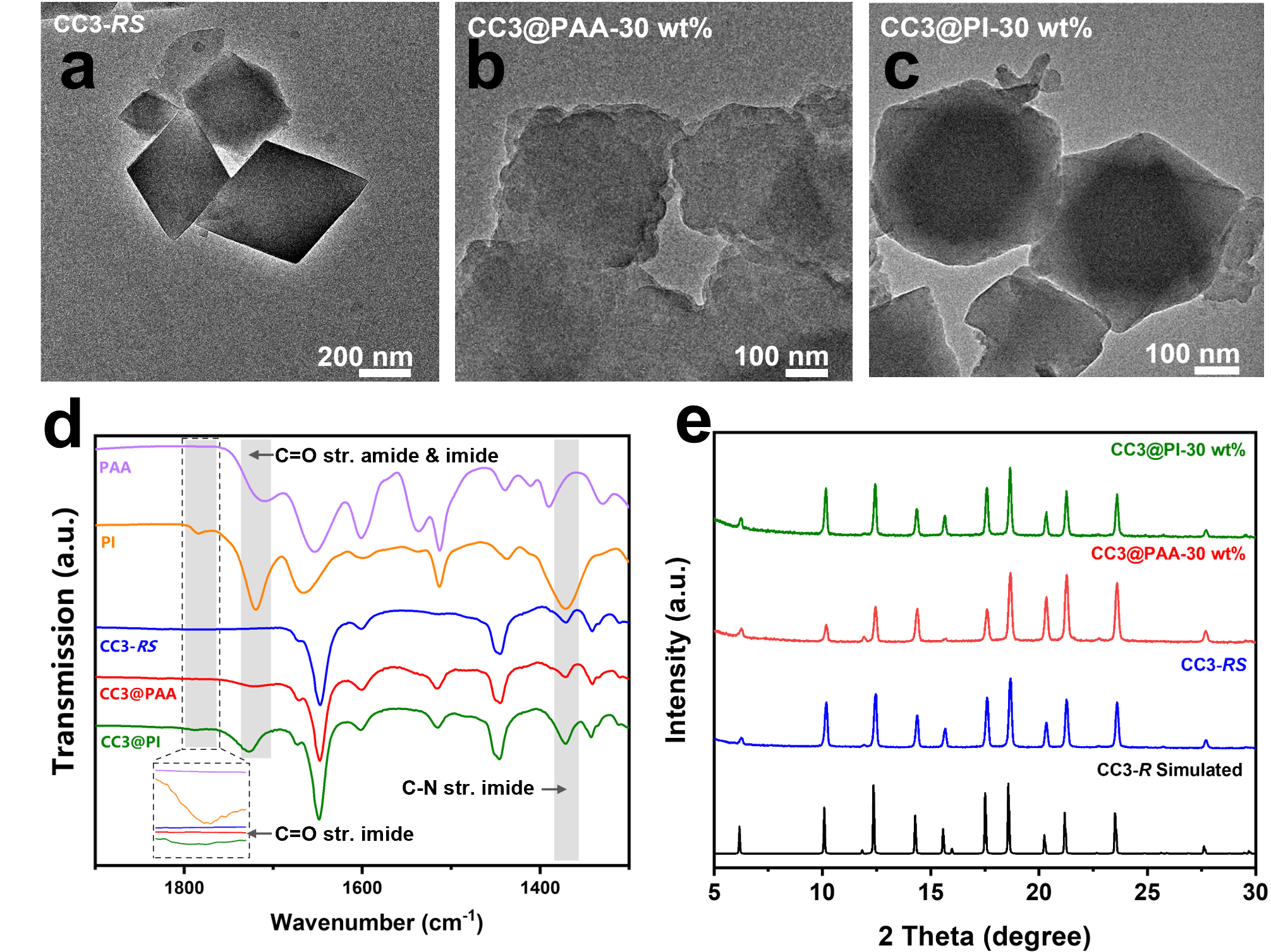


**Figure S12.** SEM images of CC3-*RS* (a), CC3@PAA-30 wt% (b) and CC3@PI-30 wt% (c). (d) FT-IR spectra of neat PAA (purple), neat PI (yellow), CC3-*RS* (blue), CC3@PAA (red) and CC3@PI (green). The CC3@PAA composite exhibited a characteristic absorption band at 1723 cm^-1^, corresponding to the C=O stretching of the amide group, in agreement with the spectrum of neat PAA. After chemical imidization, the CC3@PI composite displayed two distinct peaks at 1785 cm^-1^ and 1385 cm^-1^, which are attributed to the symmetric C=O stretch of the imide group and C-N stretching, respectively. (e) PXRD patterns comparison of CC3-*RS*, CC3@PAA-30 wt%, CC3@PI-30 wt% with simulated CC3-*R*.


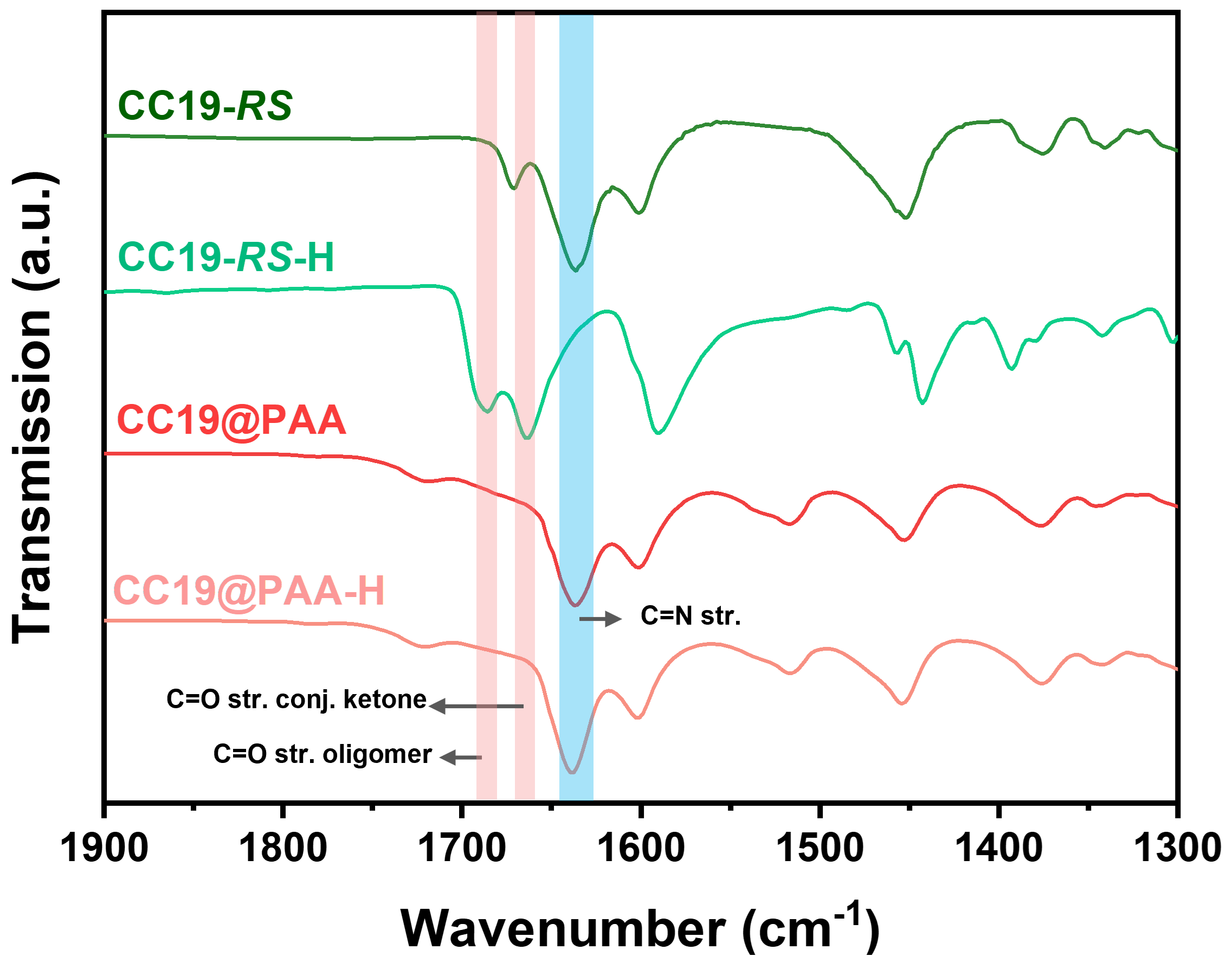


**Figure S13.** FT-IR spectra of neat CC19-*RS*, acid-treated CC19-*RS*-H, CC19@PAA and acid-treated CC19@PAA-H.

**Section S6. Gas Sorption Behaviour of POCs@polymer Core-Shell Nanostructures**

**
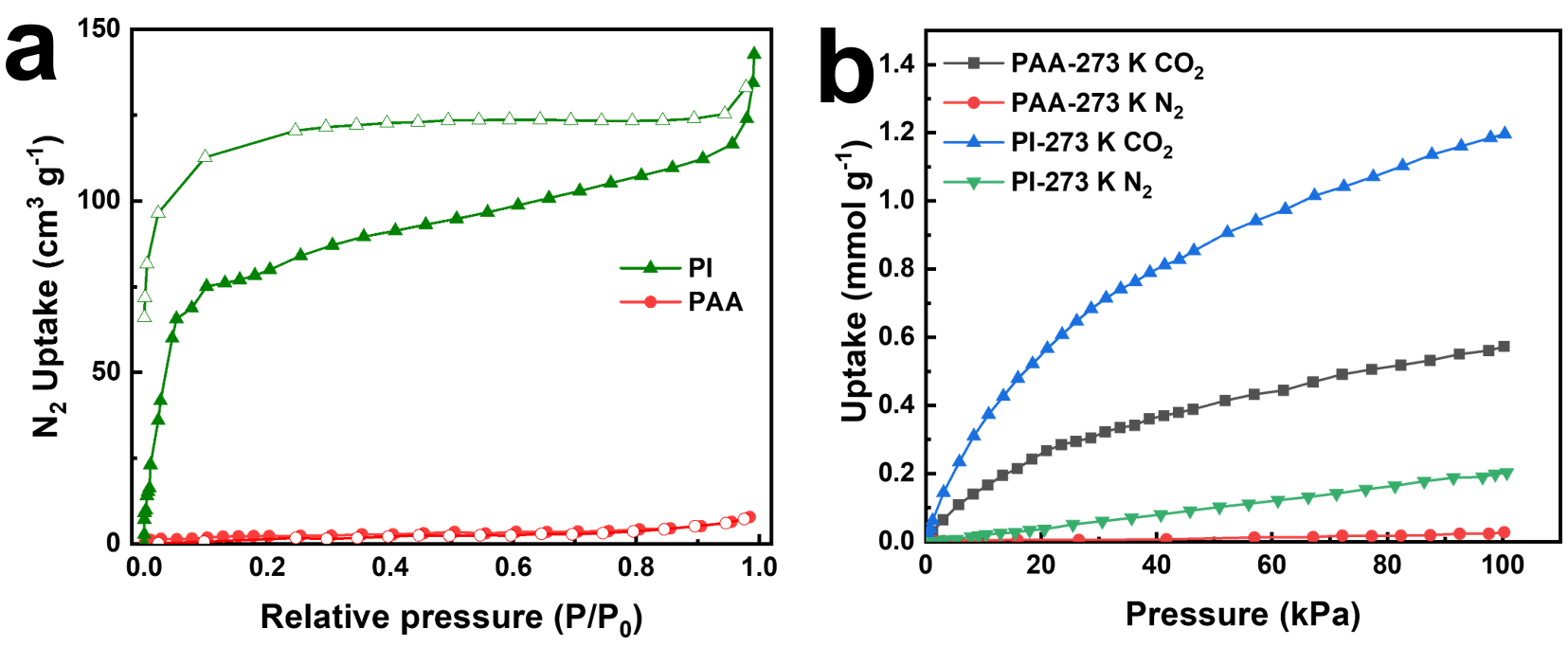
**

**Figure S14**. (a) N₂ adsorption isotherms at 77 K for PAA (red), and PI (green), PAA showed non-porous to N_2_ while PI did not. Filled and open symbols represent adsorption and desorption, respectively. (b) CO₂ and N₂ adsorption isotherms at 273 K for PAA and PI.


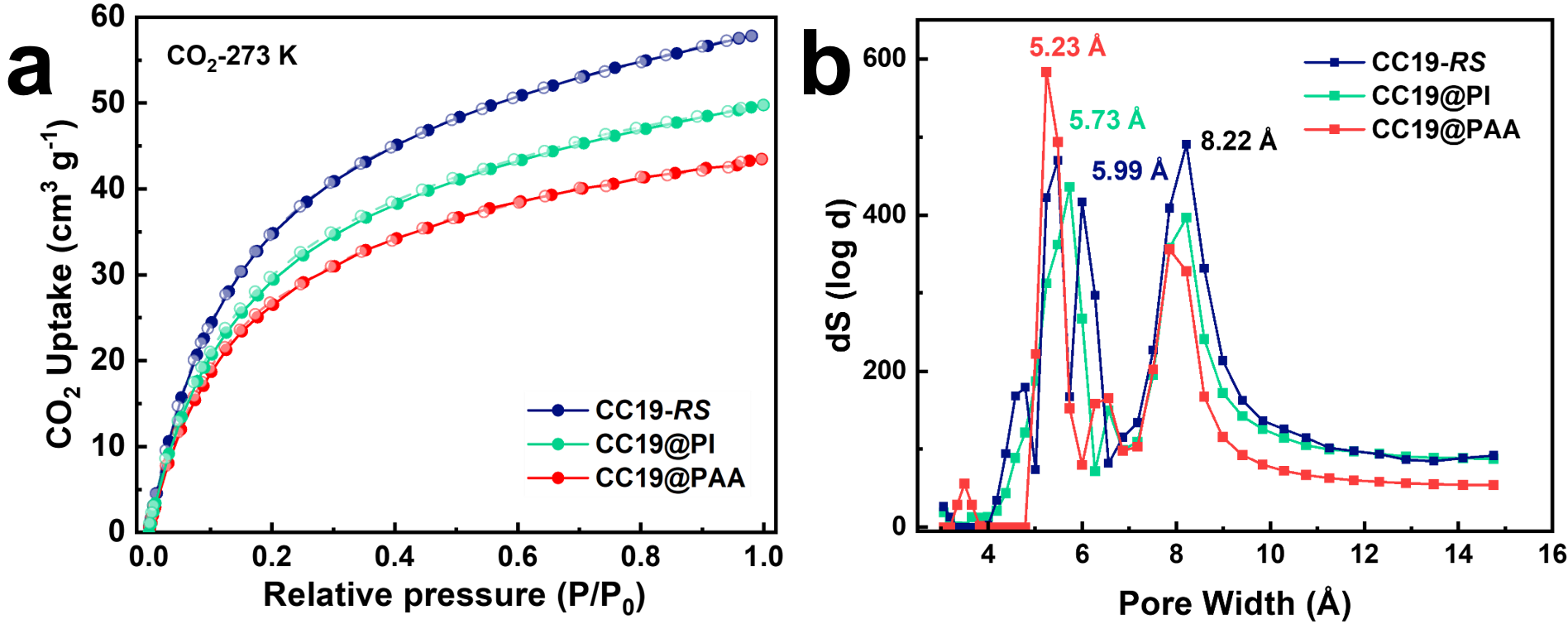


**Figure S15.** CO_2_ sorption isotherms at 273 K were measured for CC19-*RS*, CC19@PAA, and CC19@PI. Pore size distribution curves for these samples were derived from the CO_2_ adsorption data using the non-local density functional theory (NLDFT) method.


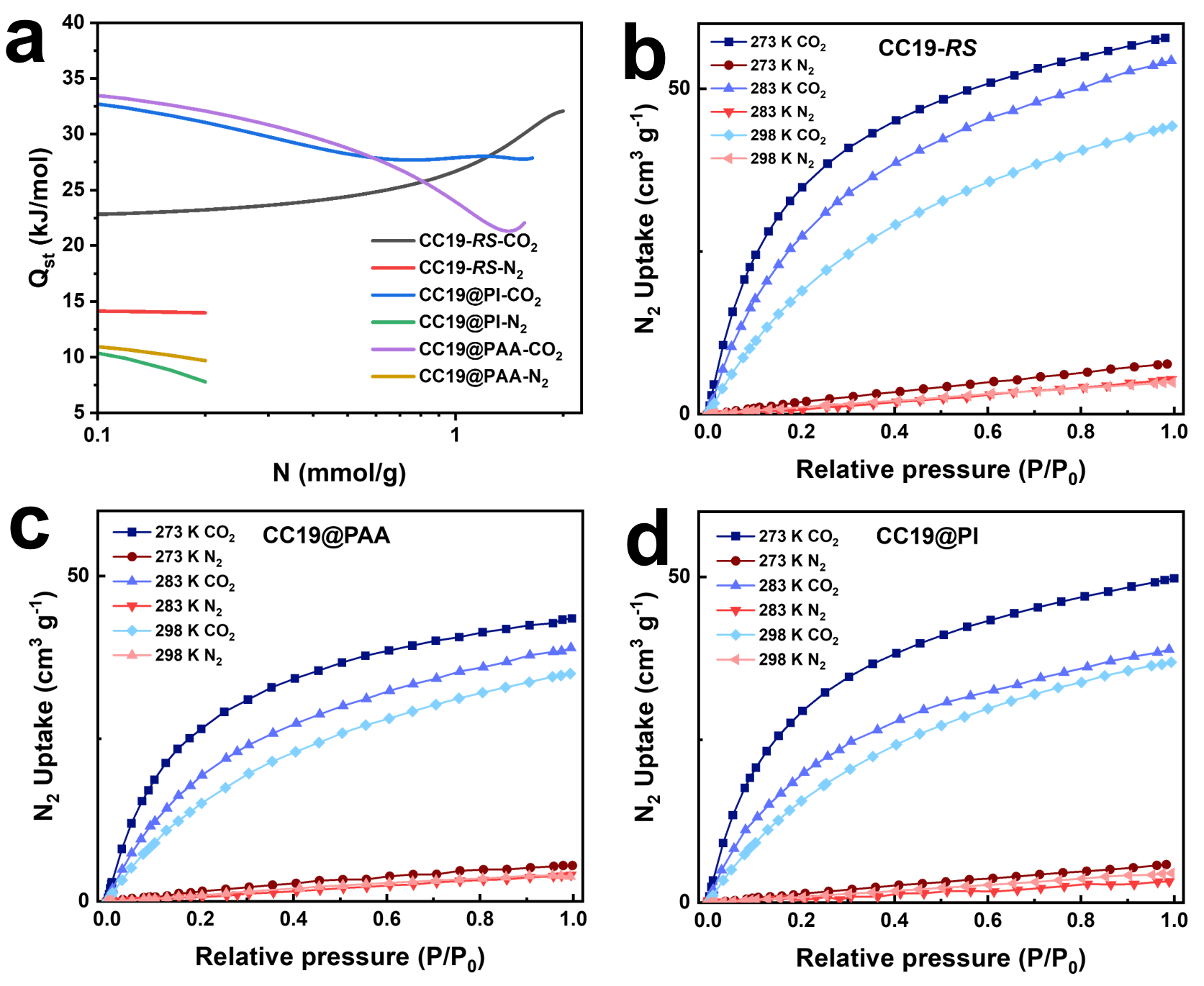


**Figure S16**. (a)Isosteric heats of adsorption of CO_2_, N_2_ for CC19-*RS*, CC19@PAA and CC19@PI calculated at 273 K, 283 K and 298 K. Sorption isotherm of CO_2_, N_2_ at 273 K , 283 K and 298 K for CC19-RS (b), CC19@PAA (c) and CC19@PI (d).

**
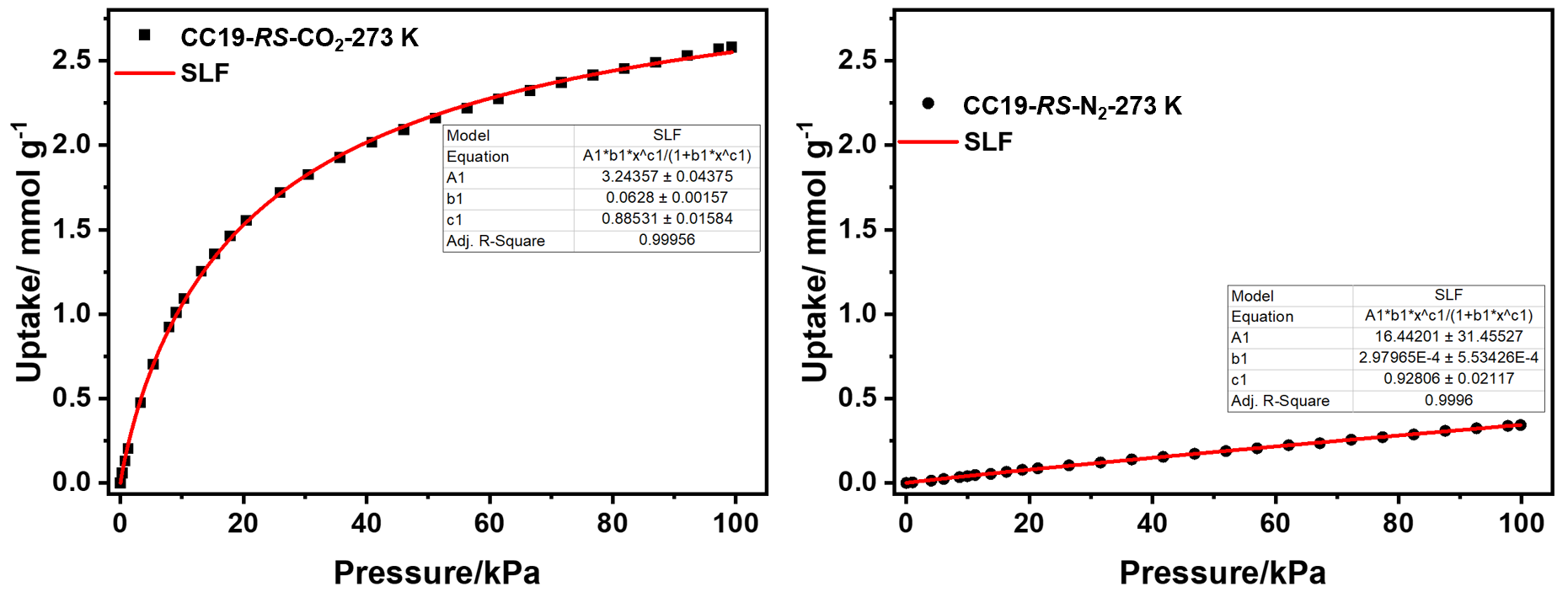
**

**Figure S17**. SLF model fitted curves of CC19-*RS* for sorption of CO_2_ and N_2_ at 273 K.

**
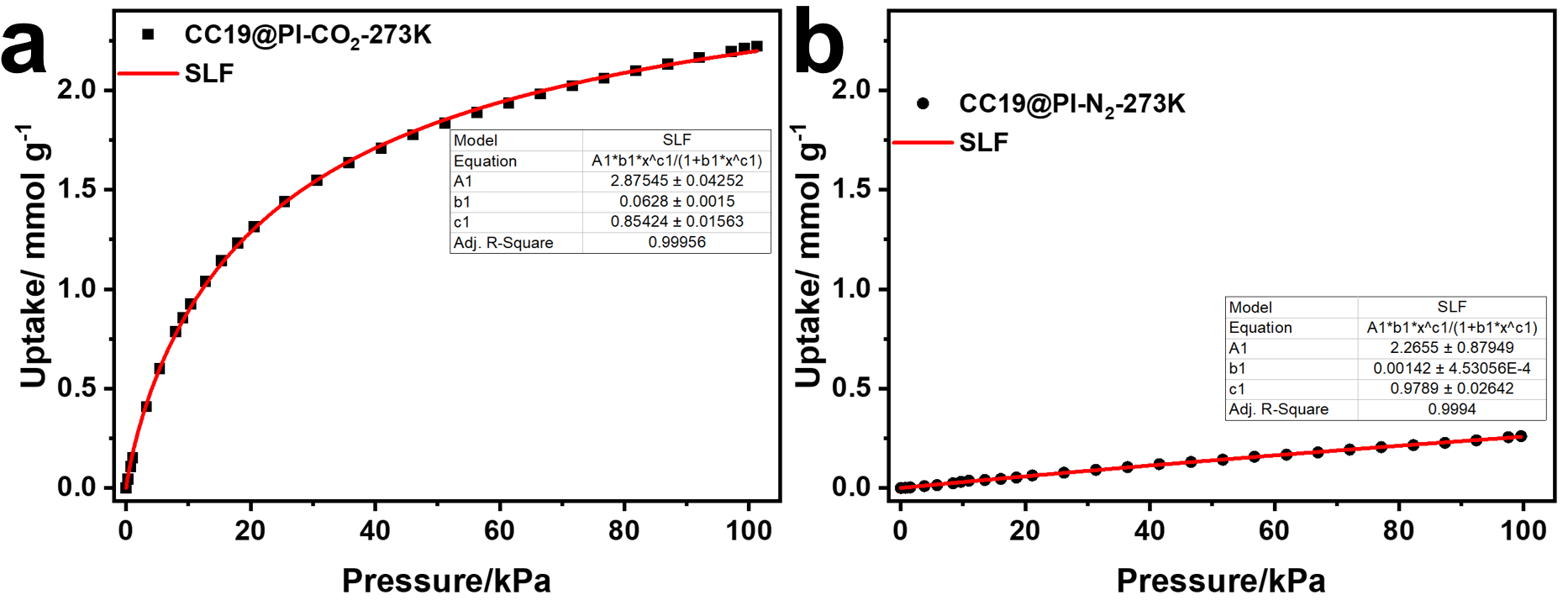
**

**Figure S18**. SLF model fitted curves of CC19@PI for sorption of CO_2_ and N_2_ at 273 K.

**
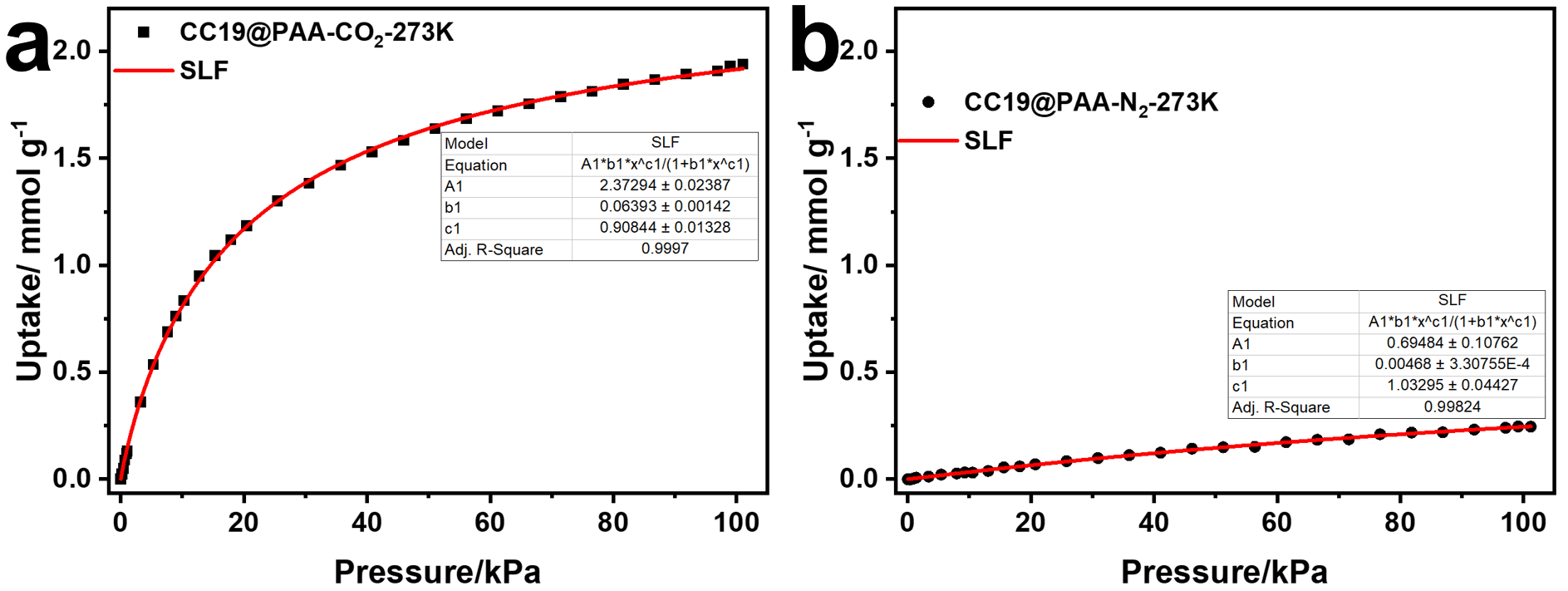
**

**Figure S19**. SLF model fitted curves of CC19@PAA for sorption of CO_2_ and N_2_ at 273 K.

**
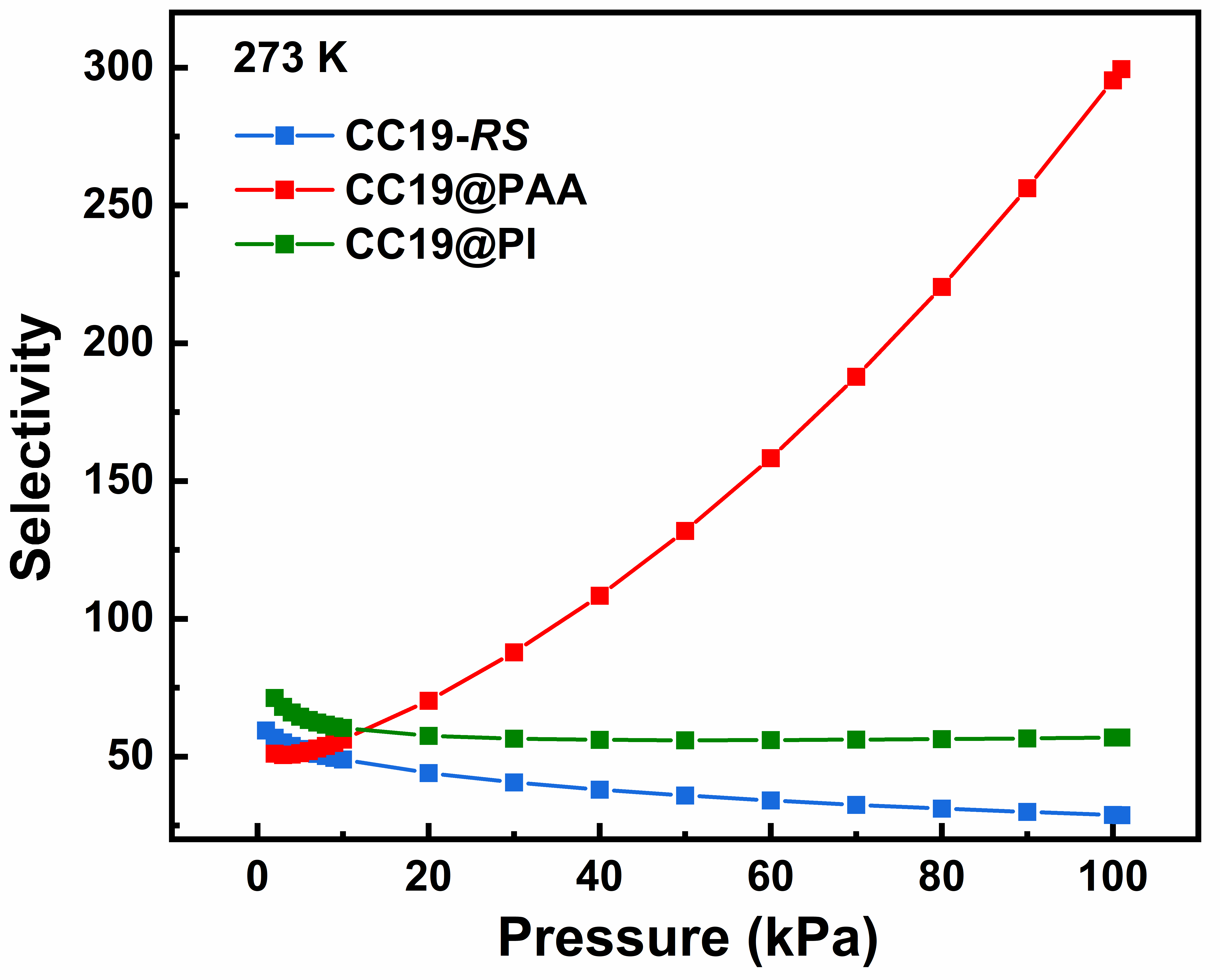
**

**Figure S20**. The calculated Ideal Adsorbed Solution Theory (IAST) selectivity of CO_2_/N_2_ (50:50, 273 K) for CC19-*RS*, CC19@PI, and CC19@PAA.


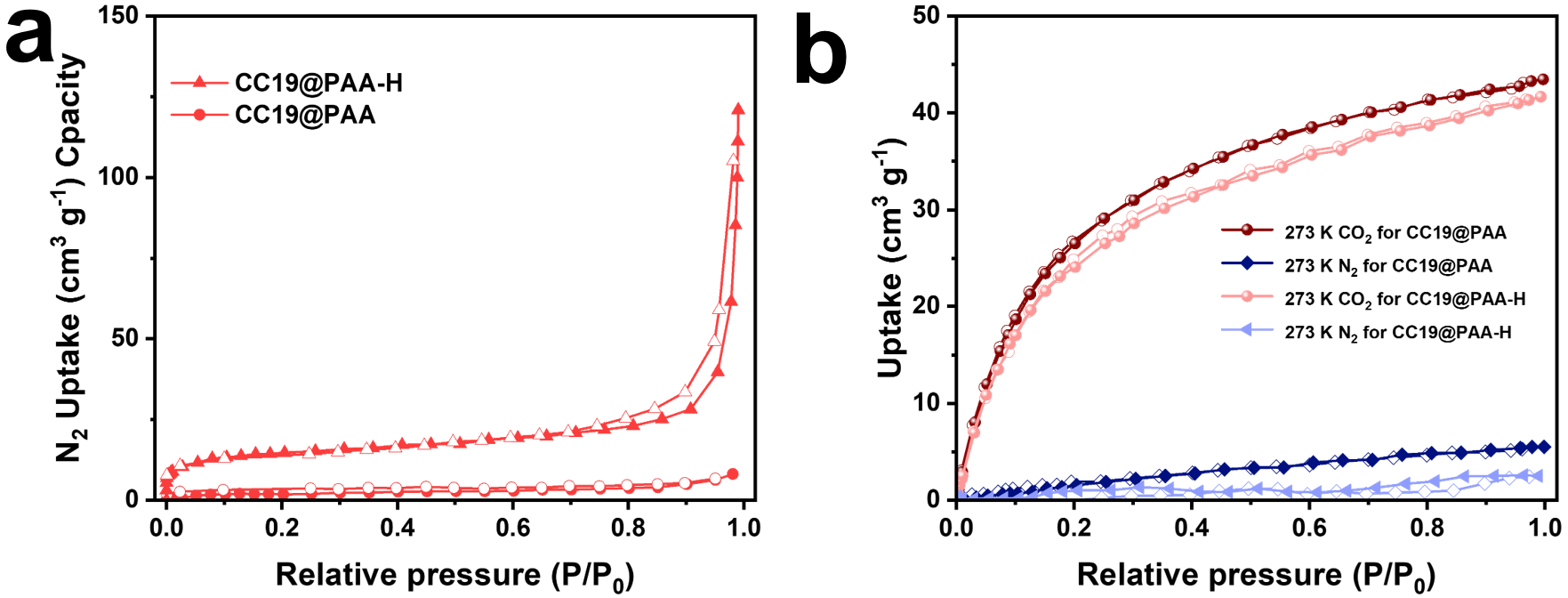


**Figure S21.** The comparison of gas sorption for CC19-*RS* and CC19@PAA before and after acid treatment. (a) N_2_ adsorption isotherms at 77 K for CC19@PAA before and after acid treatment (CC19@PAA-H). (b) Comparison of CO_2_ and N_2_ at 273 K CC19@PAA before and after acid treatment, indicating that the separation functionality is largely preserved.

**Section S7. Vapor Sorption Behaviour of POCs@polymer Core-Shell Nanostructures**

**
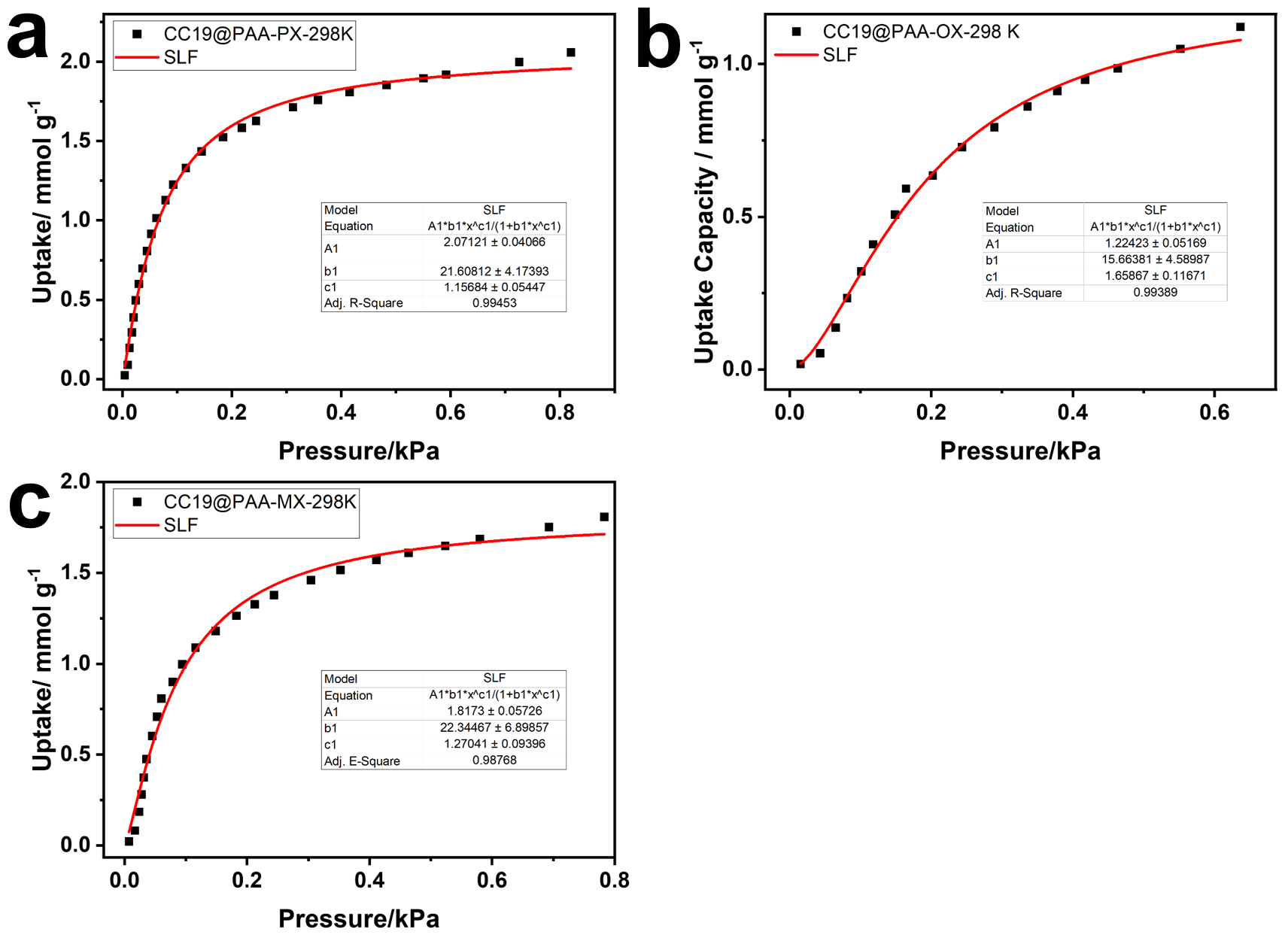
**

**Figure S22**. Xylene sorption isotherms of CC19@PAA for (a) p-xylene, (b) o-xylene, and (c) m-xylene at 298 K, fitted using the SLF model.

**
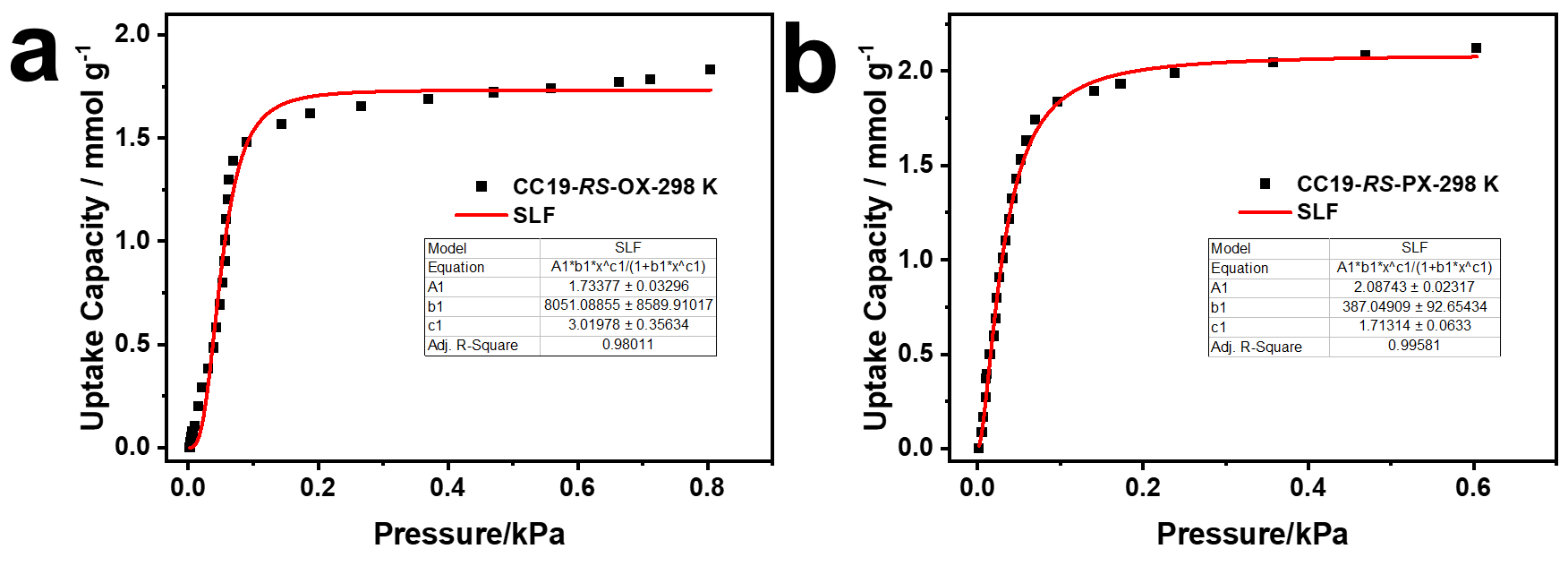
**

**Figure S23**. SLF model fitted curves of CC19-*RS* for sorption of (a) OX and (b) PX at 298 K.

**
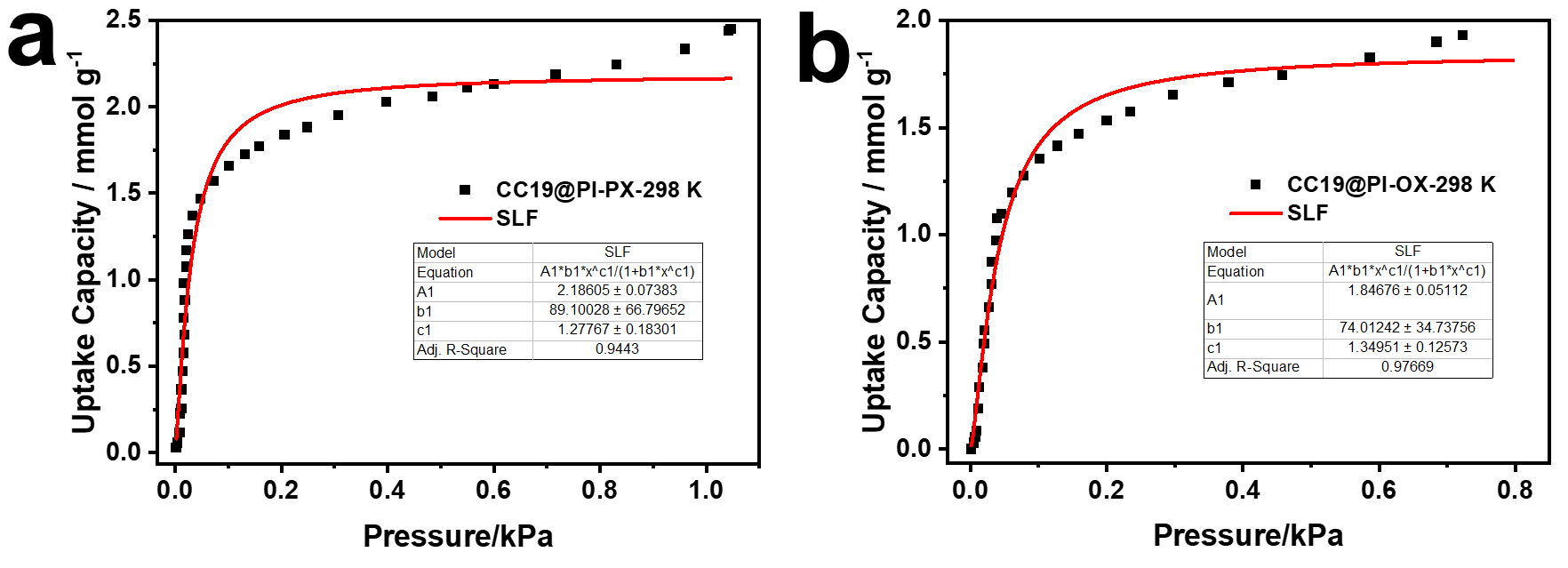
**

**Figure S24**. SLF model fitted curves of CC19@PI for sorption of (a) PX and (b) OX at 298 K.

**Section S8. Vapor Sorption Behaviour of POCs@polymer Core-Shell Nanostructures**

Table S2 The calculated CO₂/N₂ selectivity based on IAST shows that CC19@PAA delivers competitive performance relative to a variety of benchmark porous materials. A summary of the gas sorption meaurment conditions is provided below.


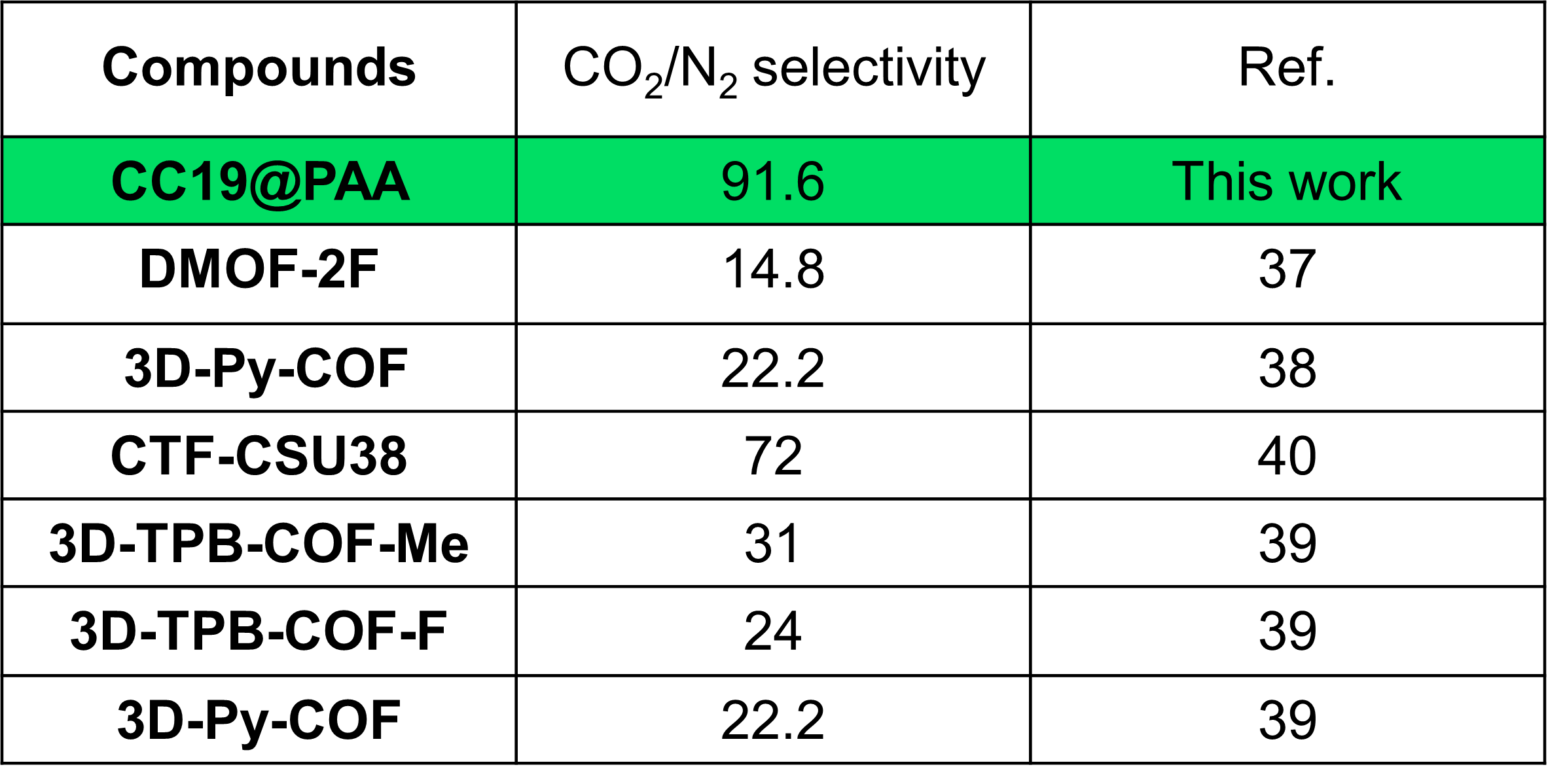


* All reference are determined for 15/85 CO_2_/N_2_ v/v mixture at 273K and 1 bar.

**Reference.**

[1] M. Petryk, J. Szymkowiak, B. Gierczyk, G. Spólnik, Ł. Popenda, A. Janiak, M. Kwit, *Org. Biomol. Chem.* **2016**, *14*, 7495-7499.

[2] T. Hasell, S. Y. Chong, K. E. Jelfs, D. J. Adams, A. I. Cooper, *J. Am. Chem. Soc.* **2012**, *134*, 588-598.

[3] Z. L. Shi, Y. Tao, J. S. Wu, C. Z. Zhang, H. L. He, L. L. Long, Y. J. Lee, T. Li, Y. B. Zhang, *J. Am. Chem. Soc.* **2020**, *142*, 2750-2754.
